# USP43 promotes gemcitabine resistance by regulating cholesterol homeostasis through E2F1 stabilization in bladder cancer

**DOI:** 10.1186/s13046-025-03621-2

**Published:** 2025-12-23

**Authors:** Mingxing Li, Tianyun Liu, Jiageng Shi, Fenfang Zhou, Zhao Deng, Yongwen Luo, Sheng Tu, Wenyu Jiang, Gang Wang, Kaiyu Qian, Yi Zhang, Yu Xiao, Xinghuan Wang, Tongzu Liu, Lingao Ju

**Affiliations:** 1 Department of Urology, Hubei Key Laboratory of Urological Diseases, Zhongnan Hospital of Wuhan University, Wuhan, 430071 China; 2 Department of Oncology, Second Affiliated Hospital of Nanchang University, Nanchang, 330006 China; 3 Department of Radiology, Zhongnan Hospital of Wuhan University, Wuhan, 430071 China; 4 Department of Biological Repositories, Department of Laboratory Medicine, Laboratory of Precision Medicine, Human Genetic Resources Preservation Center of Hubei Province, Zhongnan Hospital of Wuhan University, Wuhan, 430071 China; 5 Institute for Human Genetics and Molecular Medicine, Chinese Institutes for Medical Research, Beijing, 100069 China; 6 School of Basic Medical Sciences, Capital Medical University, Beijing, 100069 China; 7 Wuhan Research Center for Infectious Diseases and Cancer, Chinese Academy of Medical Sciences, Wuhan, 430071 China; 8 Medical Research Institute, Frontier Science Center for Immunology and Metabolism, Wuhan University, Wuhan, 430071 China

**Keywords:** E2F1, USP43, Deubiquitination, Gemcitabine resistance, Bladder cancer

## Abstract

**Background:**

Gemcitabine (GEM) is a standard treatment for bladder cancer (BLCA), but resistance to this chemotherapy often reduces its efficacy. Thus, to uncover the molecular mechanisms of this resistance is essential to reverse GEM resistance.

**Methods:**

To explore the role of the USP43/E2F1/NSDHL pathway in GEM resistance in BLCA, we employed integrated analytical approaches including gene expression profiling, biochemical assays, xenograft models, among others.

**Results:**

We discovered that the oncogene E2F1, which is highly expressed in GEM-resistant BLCA cells, plays a significant role in promoting resistance to the drug. We found that E2F1 enhances GEM resistance by activating NSDHL, an enzyme crucial for cholesterol biosynthesis, leading to increased cholesterol levels. We also identified USP43 as the deubiquitinase that stabilizes E2F1, contributing to its increased expression in response to GEM.

**Conclusion:**

Our study indicates the USP43/E2F1/NSDHL pathway as a key mechanism in cholesterol-mediated GEM resistance in BLCA, providing novel insights for therapeutic intervention to counteract chemoresistance.

**Supplementary Information:**

The online version contains supplementary material available at 10.1186/s13046-025-03621-2.

## Background

Bladder cancer (BLCA) ranks as the ninth most frequent malignancy globally, with approximately 614 thousand new cases and 220 thousand deaths reported in 2022 [1]. At the time of initial diagnosis, non-muscle-invasive bladder cancer (NMIBC) represents 70–75% of all BLCA cases, while muscle-invasive bladder cancer (MIBC) accounts for 20–25%. The primary treatment for NMIBC includes transurethral resection of the bladder tumor (TURBT), followed by adjuvant intravesical chemotherapy using agents like gemcitabine (GEM), mitomycin, or epirubicin [2]. For MIBC, the GC (GEM + cisplatin) chemotherapy regimen combined with radical cystectomy is the most commonly used first-line treatment [3]. Under these treatments, BLCA cells may develop acquired resistance to chemotherapy, leading to reduced treatment effectiveness and increasing the risk of tumor recurrence and disease progression [4]. Despite its widespread use as a primary chemotherapeutic agent for BLCA treatment, GEM faces significant challenges due to both intrinsic and acquired resistance mechanisms that limit its effectiveness. Thus, a detailed understanding of the mechanisms driving GEM resistance is crucial to identifying new therapeutic targets, enhancing treatment responses, and ultimately improving outcomes for BLCA patients.

Metabolic reprogramming is increasingly recognized as a defining feature of cancer, with BLCA showing distinct changes through both experimental and clinical studies. For instance, JMJD1A promotes BLCA progression by enhancing glycolysis via HIF1α activation [5]. Melatonin’s anti-tumor effects stem from its ability to inhibit PPARγ/ENO1-dependent glycolysis in BLCA [6], and DHCR7 promotes invasion and metastasis by altering cholesterol synthesis [7]. Evidence also highlights the significant role of dysregulated lipid metabolism in cancer progression and resistance to therapy. E2F1, an important transcription factor and well-studied member of the E2F family [8], influences several cellular processes including cell cycle progression, apoptosis, tumorigenesis, drug resistance, cell metabolism, and DNA repair [9–14]. E2F1 also affects lipid metabolism, activating genes involved in lipid synthesis, including *ACACA*, *FASN*, and *SCD1* [15, 16], and repressing CPT2 expression, which contributes to creating a lipid-rich microenvironment that supports cancer growth [17].

Cholesterol, a crucial component of lipids, plays multiple roles in cellular physiology, including membrane fluidity, signal transduction, and drug efflux, processes implicated in chemoresistance. In non-small cell lung cancer, the EGFR/Src/ERK signaling cascade is activated by cholesterol, driving resistance to EGFR tyrosine kinase inhibitors and triggering the re-expression of ERRα [18]. NSDHL is a key metabolic enzyme involved in cholesterol biosynthesis. NSDHL is highly expressed and correlates with poor prognosis in patients with breast cancer [19, 20]. Inactivation of NSDHL inhibits the growth of skin tumors and antagonizes the development of pancreatic ductal adenocarcinoma [21, 22]. However, the role of cholesterol homeostasis in BLCA remains to be fully elucidated. An in-depth investigation of the specific mechanisms of cholesterol metabolism may offer novel therapeutic avenues for overcoming GEM resistance in BLCA treatment.

USP43, part of the ubiquitin-specific protease (USP) family, functions as a critical deubiquitinase by removing ubiquitin chains from target proteins, thereby maintaining their stability and modulating their biological activity through post-translational modification. USP43 drives tumorigenesis in cancers such as colorectal [23], breast [24], and ovarian [25] cancers. Our previous investigation established that USP43 promotes metastasis in BLCA by stabilizing MYC [26]. Nevertheless, the potential role of USP43 in BLCA awaits further elucidation. Herein, we demonstrated that E2F1 is induced by GEM and plays a vital role in BLCA by directly transcriptionally activating NSDHL to modulate cholesterol levels and promote GEM resistance. However, USP43 interacts with E2F1 to promote its deubiquitination and is indispensable for the GEM-induced upregulation of E2F1. In summary, this study found that USP43 enhances GEM resistance and regulates cholesterol homeostasis in BLCA through the stabilization of E2F1, offering a new approach to counter GEM resistance in BLCA.

## Methods

### Human tissue samples

The research was carried out with the endorsement of the Medical Ethics Committee of Zhongnan Hospital of Wuhan University (approval number: 2024013K). Human BLCA tissues and paired adjacent tissues were collected from Zhongnan Hospital of Wuhan University with informed consent. BLCA tissue microarrays (HBlaU079Su01 and HBlaU108Su01) were obtained from Outdo Biotech (Shanghai, China).

### Cell culture

T24 (CSTR: 19375.09.3101HUMTCHu55), 293T (CSTR: 19375.09.3101HUMGNHu17), and UM-UC-3 (CSTR: 19375.09.3101HUMTCHu217) cell lines were obtained from and authenticated by the Chinese Academy of Sciences Cell Bank (Shanghai, China). T24 cells were cultured in RPMI 1640, 293T cells in DMEM, and UM-UC-3 cells in MEM. All media were supplemented with 10% fetal bovine serum. Authentication, confirming mycoplasma-free status, was performed using short tandem repeat (STR) profiling.

### SiRNAs and plasmids

Small interfering RNAs (siRNAs) were obtained from GenePharma (Shanghai, China). GE Healthcare Dharmacon provided the human DUBs siRNA library (G-104705). The sequences for the siRNA were:

5’-GGACUCUUCGGAGAACUUUTT-3’ (siE2F1-1);

5’-GCGCAUCUAUGACAUCACCTT-3’ (siE2F1-2);

5’-GCAAGAUGAAGUUCGUGAUTT-3’ (siNSDHL-1);

5’-GUCGAUAUCAAGAAUGGAATT-3’ (siNSDHL-2);

5’-GGUGGUCCUUUGGAUCCAATT-3’ (siUSP43-1);

5’-CCAGUUACCCGCUGGACUUTT-3’ (siUSP43-2);

The Flag-USP43 plasmid was kindly supplied by Professor Yongfeng Shang (Peking University, China). The NSDHL overexpression plasmid and the luciferase reporter plasmid used for assessing E2F1 transcriptional activity were purchased from the MiaoLing Plasmid Platform (Wuhan, China). All other constructs were generated through molecular cloning, with sequence fidelity validated by DNA sequencing.

### RNA extraction and quantitative reverse transcription PCR (qRT-PCR)

Total RNA was extracted using the HiPure Total RNA Mini Kit (R4111-03, Magen), and then reverse transcription was performed to obtain cDNA according to the instructions of a reverse transcription kit (FSQ-101, TOYOBO). Finally, cDNA was analyzed by qRT-PCR using iTaq Universal SYBR Green Supermix (1725125, Bio-Rad). The qRT-PCR primers used in this study are listed in Supplementary Table S1.

### Western blot analyses

In general, cells were lysed on ice with a mixture of RIPA solution containing PMSF and protease inhibitors and centrifuged at 12,000 rpm, and the supernatant was aspirated. Subsequently, 5× loading buffer was added to denature the protein by heating at 100 °C for 10 min. Following electrophoretic separation, the total protein was electrotransferred onto a PVDF membrane, which was then underwent blocking with 5% skim milk. The membrane was subsequently incubated with primary and secondary antibodies. The protein signal was detected by chemiluminescence eventually using a gel imager (BioSpectrum 515 Imaging System, UVP). The primary antibodies used in this study are listed in Supplementary Table S2.

### Cholesterol content measurement

BLCA cells (5 × 10^6^) were collected to extract total cholesterol, which was subsequently measured using the Total Cholesterol Assay Kit (KTB2220, Abbkine) per manufacturer’s instructions.

### Cell viability assay

Cell viability was determined via MTT assays. Cells (3000 cells/well) were seeded in 96-well plates. Following the indicated treatment, each well received 20 µL of MTT (methyl thiazolyl tetrazolium, Sigma) reagent with incubation at 37 °C for 4 h. After supernatant removal, formazan precipitates underwent solubilization in 200 µL dimethyl sulfoxide (DMSO). After gentle agitation to ensure homogeneity, absorbance was quantified at 570 nm using a microplate reader (SpectraMax M2, USA).

### Colony formation assay

The cells were seeded in 6-well plates at 3000 cells/well for approximately 10 days, then fixed with 4% paraformaldehyde and stained with 0.1% crystal violet.

### Cell apoptosis assay

The apoptosis assay was performed using the Annexin V-FITC Apoptosis Detection Kit (A5001-02 A, Simu Biotech) per manufacturer’s protocol. Briefly, treated BLCA cells were harvested and centrifuged. After discarding the supernatant, the cell pellet was gently washed with PBS and resuspended in 100 µL of 1× Annexin V binding buffer. Cells were stained with 5 µL of Annexin V-FITC and 5 µL of propidium iodide (PI), then incubated for 15 min in the dark. Finally, 400 µL of Annexin V binding buffer was added to the mixture with gentle mixing, and the cell apoptotic rates were quantified via a flow cytometer (Beckman, USA).

### Multidrug resistance assay

We used the EFLUXX-ID Green Multidrug Resistance Assay Kit (ENZ-51029, Enzo) to measure the efflux activity of major ABC transporters (MDR1, MRP, and BCRP). The assay utilizes a hydrophobic, non-fluorescent substrate that enters cells readily and is hydrolyzed by intracellular esterases into a fluorescent dye, which is subsequently extruded by active ABC transporters. Therefore, the intracellular mean fluorescence intensity (MFI) inversely reflects ABC transporter efflux activity. The assay was conducted following the manufacturer’s instructions. In brief, after treated with specific inhibitor for 5 min at 37 °C, the substrate was added to the cells, and incubation was continued for an additional 30 min at 37 °C. Flow cytometry was then used to quantify MFI.

### Cell ferrous iron assay

The intracellular ferrous iron (Fe^2+^) level was measured using a Cell Ferrous Iron Colorimetric Assay Kit (E-BC-K881-M, Elabscience). The cells were seeded in 6-well plates and transfected for 48 h. Then, 1 × 10^6^ cells from each sample were collected for the assay, which was performed in accordance with the manufacturer’s instructions.

### Lipid ROS analysis assay

The cells were seeded in 6-well plates. After 24 h of transfection, the cells were treated with or without 2 µM Erastin for 24 h. Then we added the Lipid Peroxidation Probe BODIPY-C11 581/591 (L267, Dojindo) to each plate. The cells were incubated with the probe at 37 °C for 30 min in the dark. After washing with PBS, the level of fluorescence of the cells were quantified via a flow cytometer (Beckman, USA).

### Animal studies

We established seven T24 lentiviral stably transfected cell lines (NC, shUSP43, E2F1, shUSP43 + E2F1, shE2F1, NSDHL, and shE2F1 + NSDHL), lentiviruses were obtained from GenePharma (Shanghai, China), and puromycin (Sigma, 1 µg/mL) was used to screen lentivirally transfected cells. The animal study was approved by the Experimental Animal Welfare Ethics Committee, Zhongnan Hospital of Wuhan University (approval number ZN2023147). Four-week-old male nude mice (BALB/c-nude) were acquired from Beiente BioTechnology (Wuhan, China). Following one week of acclimatization, random group assignment was implemented. A total of 1 × 10^7^ T24 stably transfected cells were injected subcutaneously into the mice by resuspension in 100 µL of sterile PBS. The volume of the subcutaneous tumors was measured every five days with Vernier calipers until the endpoint. The tumor volume was measured via the following formula: V = 1/2 × L × S^2^ (L: long diameter, S: short diameter). Beginning 10 days post-tumor inoculation, the nude mice received weekly intraperitoneal injections of either PBS or 50 mg/kg GEM. No blinding was employed for the drug treatment. At 35 days post-inoculation, the mice were euthanized, and the subcutaneous tumors were surgically excised. The tumor volume and weight were subsequently recorded. Excised tissues underwent 4% paraformaldehyde fixation, followed by hematoxylin and eosin (H&E) staining and immunohistochemical (IHC) analysis. For toxicity study, we established subcutaneous tumor-bearing nude mice and randomly assigned them to two groups. One group received intraperitoneal PBS (control), and the other received GEM according to the schedule used in xenograft models. Body weight was recorded every 5 days throughout the treatment period. At the endpoint, mice were euthanized and the heart, liver, spleen, lung, and kidney were collected for H&E staining.

### Coimmunoprecipitation assay

Co-IP assays were conducted via the BeaverBeads™ Protein A/G Magnetic Immunoprecipitation Kit (22202-100, Beaver) following the manufacturer’s protocol. Briefly, the cells were lysed to obtain the supernatant, after which the lysates were incubated overnight with the indicated antibody under constant shaking at 4 °C. On the second day, 20 µL pre-washed protein A/G magnetic beads were introduced into the antigen-antibody mixture and incubated for 2 h. The beads were subsequently washed three times with IP washing buffer to prevent nonspecific interactions. The bound proteins were eluted with 1× loading buffer and subjected to denaturation at 100 °C for 5 min. The eluted proteins were subsequently analyzed via Western blotting.

### Chromatin Immunoprecipitation (ChIP)

ChIP assays were performed following the manufacturer’s protocol. T24 cells (1 × 10^7^) were fixed with 1% formaldehyde for 10 min at 37 °C, followed by quenching with 0.125 M glycine for 5 min at 37 °C. The cells were subsequently lysed on ice in ChIP Sonication Cell Lysis Solution and sonicated to break the DNA fragments. The lysates were subsequently immunoprecipitated overnight at 4 °C with an anti-E2F1 antibody (3742, CST) or IgG (B900610, Proteintech). The next morning, the lysates were incubated for 3 h with the addition of washed protein A/G magnetic beads. Afterward, the beads were rinsed sequentially with low-salt followed by high-salt solutions to yield purified DNA for later quantitative PCR (qPCR) analysis. The ChIP-qPCR primer sequences used in this study are listed in Supplementary Table S1.

### Immunofluorescence

BLCA cells grown on coverslips underwent 4% paraformaldehyde fixation, 0.4% Triton X-100 permeabilization, and 2% BSA blocking. The samples were subsequently incubated with primary antibodies, secondary antibodies and DAPI (0.5 µg/mL) in the dark. Images of the immunofluorescence-stained samples were captured with a confocal laser microscope (Nikon C2+, Japan).

### Dual-luciferase reporter assay

Luciferase activity was quantified employing the Dual-Luciferase^®^ Assay Kit (E1910, Promega) following transfection of 293T cells carrying the indicated luciferase plasmids. Firefly luciferase activity measurements were standardized against Renilla luciferase reference values.

### Ubiquitination assay

Ubiquitination assays were carried out as described previously [26]. Briefly, following transfection of cells with the corresponding siRNA or plasmid, MG132 was added 6 h prior to harvesting, followed by lysis of the cells on ice with a mixture of RIPA solution containing PMSF and protease inhibitors. After centrifugation at 4 °C for 10 min, the supernatant was collected and subsequently incubated overnight at 4 °C with the corresponding antibody. Finally, polyubiquitinated E2F1 was detected via the previously described procedures of IP and Western blotting.

### Statistical analysis

The statistical analysis was performed via GraphPad Prism (version 9). Two-tailed Student’s t test, one-way ANOVA, Pearson’s correlation and the log-rank test of Kaplan-Meier analysis were used as appropriate. The data are presented as the means ± standard deviations (SDs). *p* < 0.05 was considered statistically significant.

## Results

### E2F1 is induced by GEM and highly expressed in BLCA

**Fig. 1:**
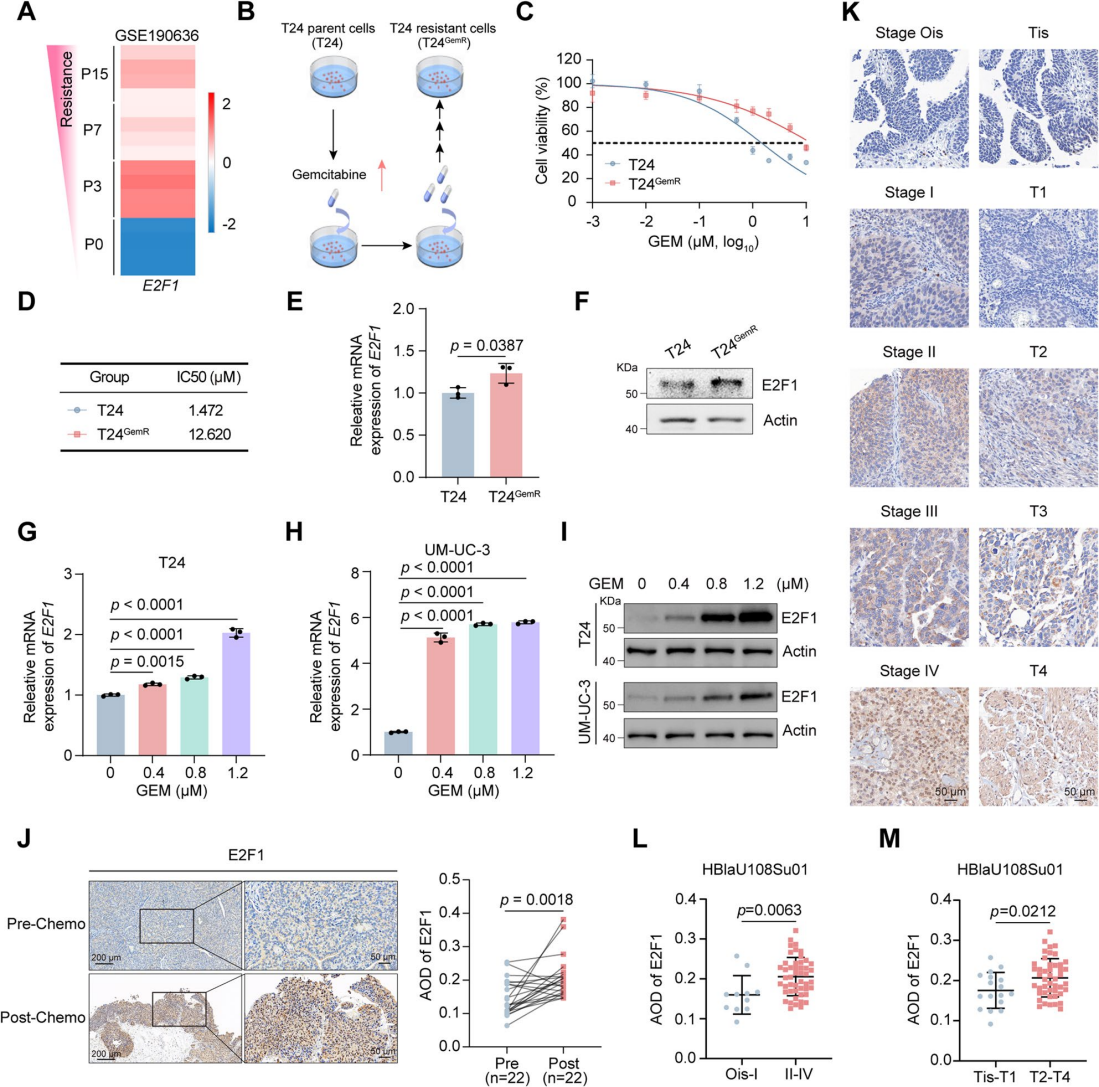
E2F1 is induced by GEM and is highly expressed in BLCA. **A** E2F1 expression levels from P0 to P15 in T24 cells in GSE190636 (RNA-seq data). **B **Schematic diagram showing the establishment of T24 GEM-resistant cell lines. **C** Cell viability assay results showing the viability of T24^GemR^ and T24 cells after 48 hrs of exposure to different concentrations of GEM (n = 5). **D** The IC50 values of T24 and T24^GemR^ cells. **E** The mRNA level of E2F1 in T24 and T24^GemR^ cells was detected via qRT-PCR (n = 3). **F** Western blot analysis of E2F1 protein expression in T24 and T24^GemR^ cells. (G-H) The mRNA level of E2F1 in T24 (**G**) and UM-UC-3 (**H**) cells after 48 hrs of exposure to different concentrations of GEM, as measured by qRT-PCR (n = 3). **I** Western blot analysis showing the protein levels of E2F1 in T24 and UM-UC-3 cells after 48 hrs of exposure to different concentrations of GEM. **J** Representative images (left panel) and statistical values (right panel) of IHC staining analysis of E2F1 protein levels in patients with BLCA treated with GEM chemotherapy (n = 22). The average optical density (AOD) = integral optical density (IOD)/positive staining area. **K** Representative images of IHC staining of E2F1 protein levels in different AJCC stages (left panel, seventh edition of the AJCC: stages Ois, I, II, III, and IV) and T stages (right panel, Tis, T1, T2, T3, and T4) from the HBlaU108Su01. **L**-**M** Statistical graphs of the results of the IHC staining analysis of E2F1 protein levels in patients with different AJCC stages (M) and T stages (**N**). Statistical significance was determined by two-tailed unpaired Student’s t test (**E**, **L**, **M**), one-way ANOVA with Dunnett’s multiple comparisons test (**G**, **H**), and two-tailed paired Student’s t test (**J**). The data are shown as the means ± SDs

To investigate the molecular mechanisms of GEM resistance in BLCA, we first analyzed RNA sequencing (RNA-seq) data from previous studies comparing GEM-resistant and parental BLCA cells [27]. Gene set enrichment analysis (GSEA) revealed a significant increase in E2F target pathways in GEM-resistant cells (Supplementary Fig. S1A-C). Given our group’s previous demonstration of the critical role of E2F1 in BLCA proliferation and metastasis [28], we sought to determine its potential involvement in GEM resistance. As shown in the heatmap, E2F1 expression was markedly higher in GEM-resistant BLCA cells than in parental cells (Fig. 1A). Subsequently, we successfully established a GEM-resistant BLCA cell line (T24^GemR^) through stepwise drug concentration escalation, which presented a more than 8-fold higher IC50 value for GEM than did parental T24 cells (Fig. 1B-D). Both the mRNA and protein levels of E2F1 were markedly elevated in T24^GemR^ cells compared with those in T24 cells (Fig. 1E-F). Notably, elevating GEM concentration dose-responsively upregulated E2F1 expression at transcriptional and translational levels in both T24 and UM-UC-3 cells (Fig. 1G-I). These findings indicate that E2F1 expression is inducible by GEM, and together with subsequent functional experiments, support its potential contribution to GEM chemoresistance. To assess the clinical relevance of E2F1 in BLCA, analysis via the GEPIA database revealed significantly higher E2F1 expression in BLCA tissues than in normal tissues (Supplementary Fig. S1D). Further evaluation of the GES13507 dataset revealed elevated E2F1 levels in BLCA tissues compared with adjacent normal tissues, with a positive correlation corresponding to the AJCC stage and T stage. Moreover, patients with high expression of E2F1 presented a poorer prognosis (Supplementary Fig. S1E-H). Furthermore, qRT-PCR analysis of the BLCA tissues from the Zhongnan Hospital of Wuhan University (ZNWU) in-house dataset revealed that E2F1 expression was notably upregulated in tumor tissues relative to paired adjacent normal tissues (Supplementary Fig. S1I). To further explore the clinical relevance of E2F1 in GEM resistance, we collected paired tumor tissues from bladder cancer patients before and after GEM-based chemotherapy (*n* = 22 pairs). Immunohistochemical analysis showed that E2F1 expression is significantly increased in post-chemotherapy tumors compared with the corresponding pre-chemotherapy samples, indicating that E2F1 upregulation is associated with GEM exposure (Fig. 1J). We then stained BLCA tissue microarrays immunohistochemically, which revealed that E2F1 protein levels displayed a progressive rise as AJCC stage and T stage advanced (Fig. 1K-M). In summary, E2F1 can be induced by GEM, and E2F1 is highly expressed and predicted poor prognosis in patients with BLCA.

### E2F1 promotes GEM resistance in BLCA in vitro and in vivo

**Fig. 2:**
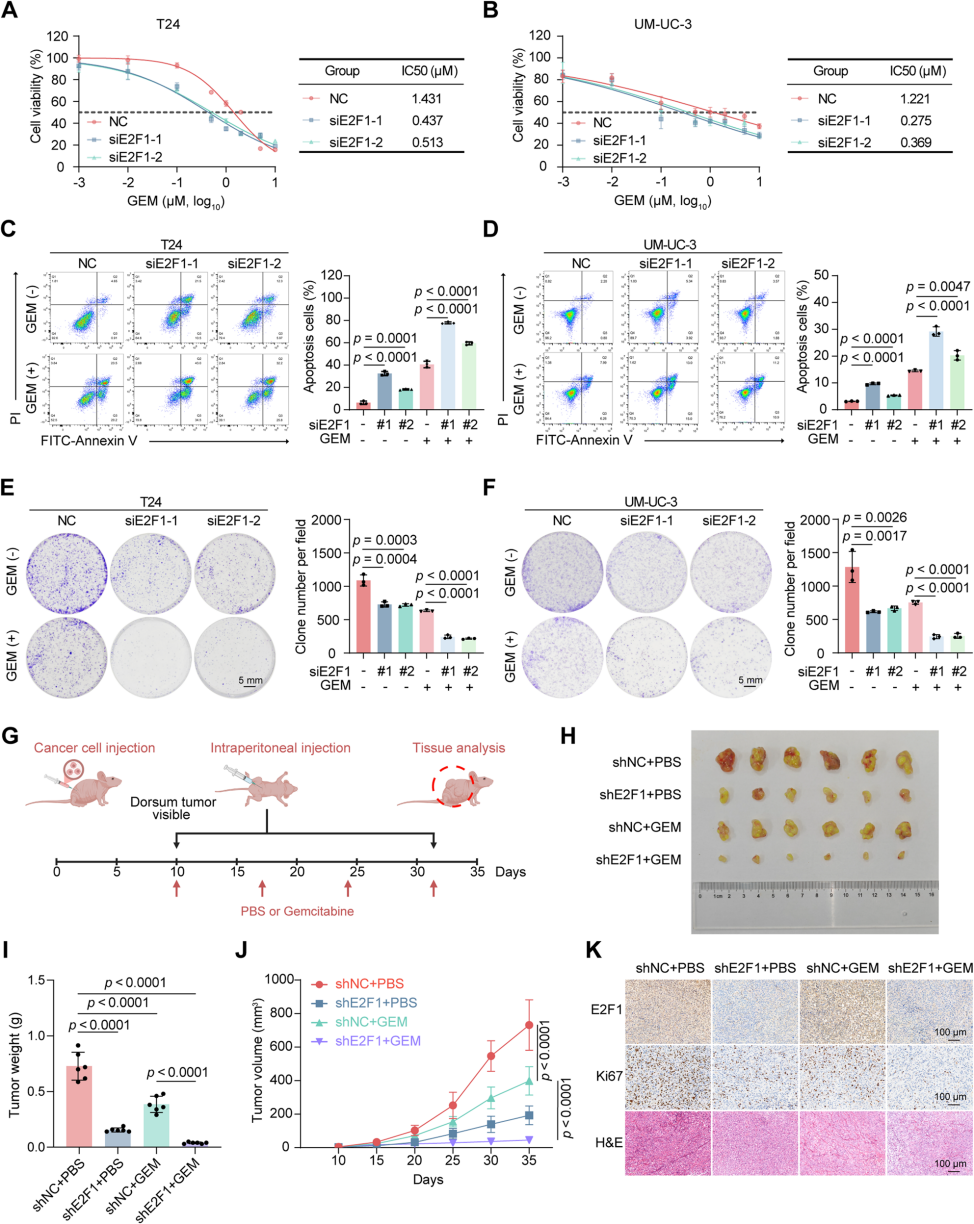
E2F1 promotes GEM resistance in BLCA in vitro and in vivo. **A**-**B** Cell viability assay showing the viability and IC50 values of T24 (A) and UM-UC-3 (B) cells with E2F1 knockdown after 48 hrs of exposure to GEM. **C**-**D** Representative images (left diagram) and statistical analysis (right diagram) of apoptosis assays in T24 (**C**) and UM-UC3 (**D**) cells with E2F1 knockdown following 1 μM GEM or PBS treatment for 48 hrs (n = 3). **E**-**F** Representative images (left diagram) and statistical analysis (right diagram) of colony formation assays in T24 (**E**) or UM-UC3 (**F**) cells with E2F1 knockdown following 1 μM GEM or PBS treatment (n = 3). **G** Schematic diagram of the establishment of the chemotherapy assay model. **H** Gross view of the subcutaneous xenograft tumors in each group (n = 6). **I** The tumor weights of each group at the endpoint (n = 6). **J** The tumor volume of each group was measured on different days (n = 6). **K** Representative images of H&E and IHC staining of subcutaneous xenograft tumors from each group. The scale bar is 100 μm. Statistical significance was determined by one-way ANOVA with Dunnett’s multiple comparisons test (**C**-**F**) and one-way ANOVA with Tukey’s multiple comparisons test (**I**, **J**). The data are shown as the means ± SDs.

Next, we investigated the role of E2F1 in regulating BLCA cell GEM resistance in vitro. We examined the effects of E2F1 on GEM resistance in T24 and UM-UC-3 cells by cell viability, cell apoptosis and colony formation assays. Our results revealed that E2F1 knockdown increased the sensitivity of BLCA cells to GEM, and under GEM treatment, the cells in the E2F1-knockdown group presented increased levels of apoptosis and formed fewer colonies (Fig. 2A-F). To further explore the effect of E2F1 on BLCA GEM resistance in vivo, stable E2F1-knockdown cell lines were generated via lentiviral transduction. The results of the xenograft models demonstrated that both E2F1 knockdown and intraperitoneal injection of GEM inhibited tumor growth, and notably, the two synergistically further inhibited tumor growth (Fig. 2G-K). We monitored potential toxicity of GEM, both by recording body weight and by assessing histopathological changes of major organs in an additional toxicity evaluation experiment using the same GEM regimen as in the tumor-bearing model. The results indicated an absence of overt systemic toxicity in nude mice at the dose and schedule used in our in vivo experiments (Supplementary Fig. S2). Overall, these results suggest that E2F1 promotes GEM resistance in BLCA in vitro and in vivo.

### E2F1 promotes GEM resistance in BLCA by modulating cholesterol homeostasis

**Fig. 3:**
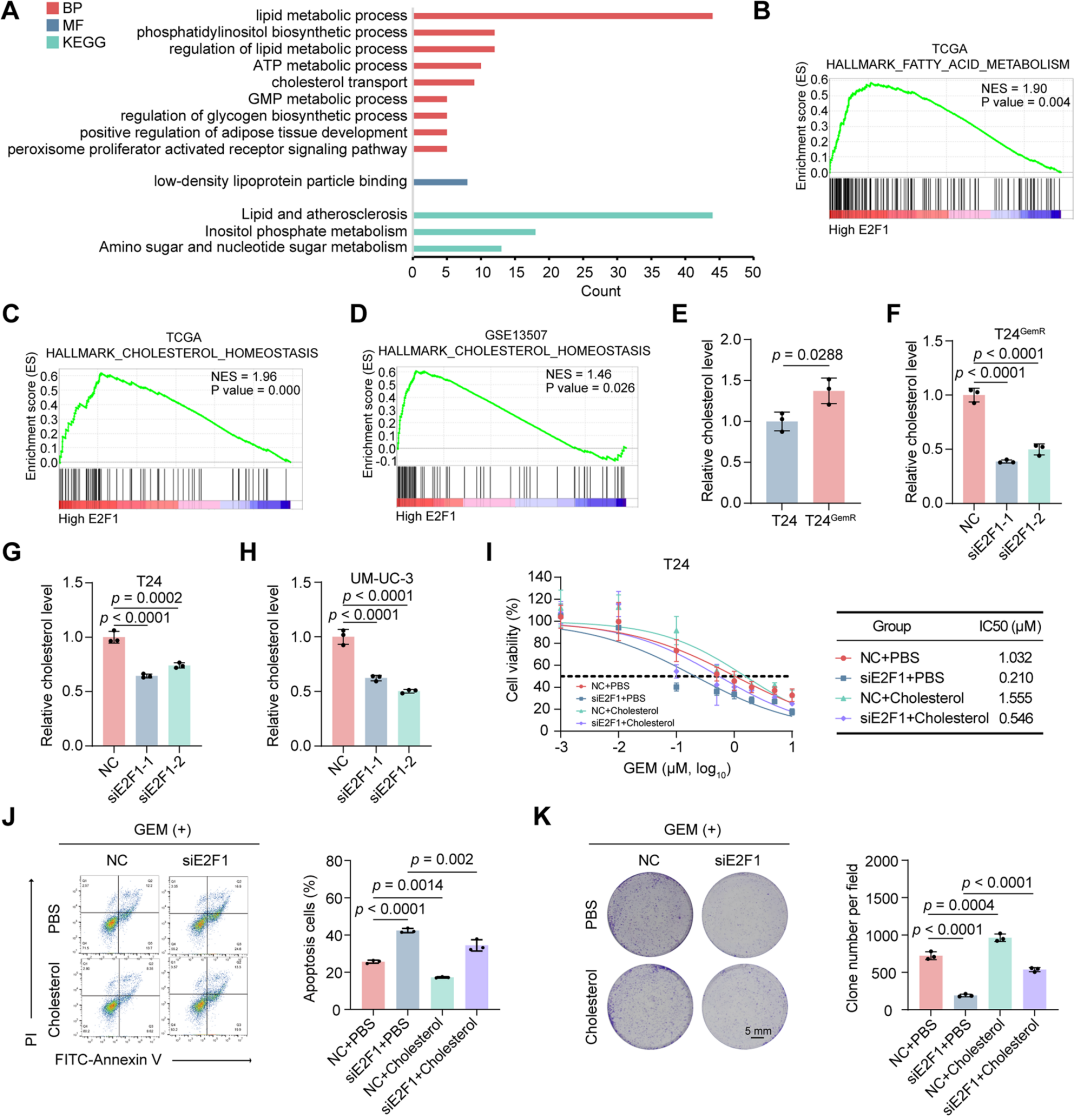
E2F1 promotes GEM resistance in BLCA by modulating cholesterol homeostasis. **A** Biological process (BP), molecular function (MF) and Kyoto Encyclopedia of Genes and Genomes (KEGG) terms related to E2F1 from enrichment analysis of the gene expression matrix from GSE190636 (RNA-seq data). **B**-**C** GSEA enrichment of the TCGA database revealed that E2F1 is positively related to the fatty acid metabolism (**B**) and cholesterol homeostasis (**C**) pathways. **D** GSEA of the GSE13507 dataset revealed that E2F1 is positively related to the cholesterol homeostasis pathway. **E **Relative cholesterol levels in T24 and T24^GemR^ cells (n = 3). **F** Relative cholesterol levels in T24^GemR^ cells after E2F1 knockdown (n = 3). **G**-**H** Relative cholesterol levels in T24 (**G**) and UM-UC-3 (**H**) cells after E2F1 knockdown (n = 3). **I** Cell viability and IC50 values of T24 cells in each group after 48 hrs of treatment with different concentrations of GEM, as detected via the MTT assay (n = 5). **J** Apoptosis analysis of T24 cells in each group after 48 hrs of treatment with 1 μM GEM (n = 3). **K** Colony formation assay of T24 cells in each group after 48 hrs of treatment with 1 μM GEM (n = 3). Statistical significance was determined by two-tailed unpaired Student’s t test (**E**), one-way ANOVA with Dunnett’s multiple comparisons test (**F**-**H**) and one-way ANOVA with Tukey’s multiple comparisons test (**J**, **K**). The data are shown as the means ± SDs

To explore the mechanism through which E2F1 promotes GEM resistance in BLCA, we performed GO and KEGG analyses of RNA-seq data from GEM-resistant and parental BLCA cells [27]. The results revealed significant enrichment of lipid metabolism-related processes such as lipid metabolism and phospholipid biosynthesis in GEM-resistant cells (Fig. 3A). Further GSEA of the TCGA BLCA dataset and GSE13507 dataset suggested that E2F1 may regulate fatty acid metabolism and cholesterol homeostasis (Fig. 3B-D). These findings collectively indicate that E2F1 potentially promotes GEM resistance through the modulation of cholesterol metabolism. We subsequently measured intracellular cholesterol levels in T24 and T24^GemR^ cells. Notably, T24^GemR^ cells presented a significantly greater cholesterol content than did T24 cells (Fig. 3E). Knockdown of E2F1 markedly reduced cholesterol levels in T24^GemR^, T24, and UM-UC-3 cells (Fig. 3F-H). Furthermore, cell viability, apoptosis, and colony formation assays demonstrated that exogenous cholesterol supplementation reversed the increased sensitivity to GEM resulting from E2F1 depletion (Fig. 3I-K). These results demonstrate that E2F1 promotes GEM resistance in BLCA by modulating cholesterol homeostasis.

### E2F1 transcriptionally activates NSDHL

**Fig. 4:**
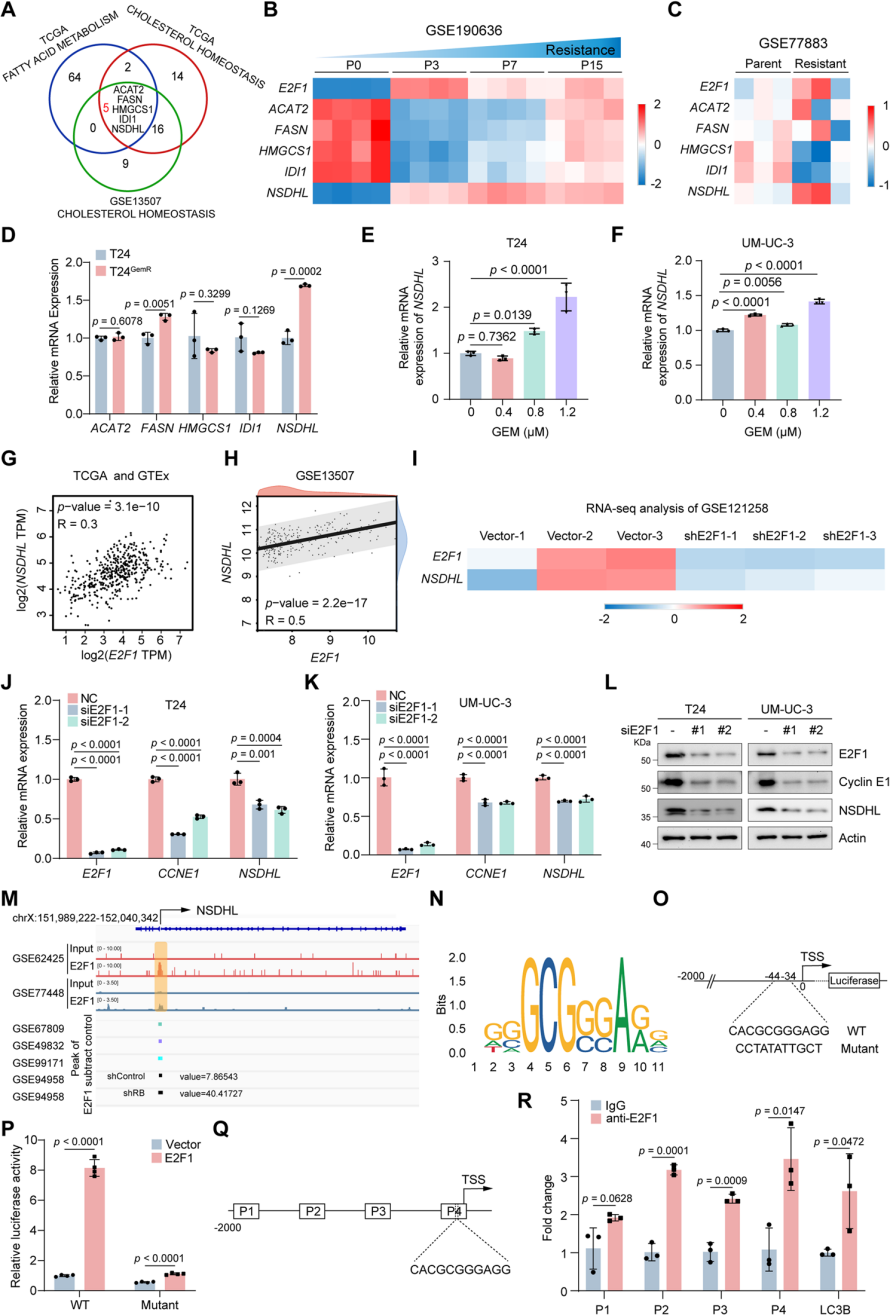
E2F1 transcriptionally activates NSDHL. **A** ACAT2, FASN, HMGCS1, IDL1 and NSDHL were identified as intersecting core genes from three enriched pathways. **B** Heatmap analysis of the expression levels of the indicated genes in GSE190636 (RNA-seq data). **C** Heatmap analysis of the expression levels of the indicated genes in GSE77883 (RNA-seq data). **D** The mRNA levels of ACAT2, FASN, HMGCS1, IDL1 and NSDHL in T24 and T24^GemR^ cells were determined via qRT-PCR (n = 3). **E**-**F** The mRNA level of NSDHL in T24 (**E**) and UM-UC-3 (**F**) cells following 48 hrs of exposure to different concentrations of GEM, as detected via qRT-PCR (n = 3). **G**-**H** Pearson’s correlation test revealed a significant positive correlation between the mRNA levels of E2F1 and NSDHL in BLCA tumor, BLCA normal and bladder tissues in GEPIA (**G**) and GSE13507 (**H**). **I** Heatmap analysis of the expression levels of E2F1 and NSDHL in GSE121258 (RNA-seq data). **J**-**K** Quantification of E2F1, CCNE1 and NSDHL mRNA expression in T24 (J) and UM-UC-3 (K) cells after E2F1 knockdown (n = 3). **L** Western blot analysis of the protein levels of E2F1, Cyclin E1, and NSDHL after E2F1 knockdown. **M** The E2F1 binding peaks in the NSDHL promoter region were identified through IGV visualization of ChIP-seq data. **N** Putative E2F1-binding motif identified in the NSDHL promoter region through JASPAR database analysis. **O **Schematic illustration of the potential sequence of E2F1 at the NSDHL promoter binding site and the constructed luciferase plasmids containing wild-type or mutant NSDHL promoter sequences. **P** Dual-luciferase reporter assay in 293T cells cotransfected with the NSDHL promoter plasmid (wild-type or mutant) and the empty vector or E2F1 overexpression plasmid (n = 4). **Q** Schematic graph of primers designed for ChIP-qPCR of the NSDHL promoter sequence. **R** ChIP-qPCR analysis showing the degree of enrichment of E2F1 in different regions of the NSDHL promoter. IgG indicates the negative control (n = 3). Statistical significance was determined by two-tailed unpaired Student’s t test (**D**, **P**, **R**), one-way ANOVA with Dunnett’s multiple comparisons test (**E**, **F**, **J**, **K**) and Pearson’s correlation (**G**, **H**). The data are shown as the means ± SDs

We further investigated the molecular mechanism by which E2F1 regulates cholesterol homeostasis. We intersected the candidate genes enriched in lipid metabolism and cholesterol homeostasis pathways identified through E2F1 expression analysis. The results revealed ACAT2, FASN, HMGCS1, IDI1, and NSDHL as potential downstream targets of E2F1 in the regulation of cholesterol homeostasis (Figs. 3B-D and 4A). Heatmap analysis revealed that among these candidates, NSDHL presented the most concordant expression pattern with E2F1 in GEM-resistant versus parental BLCA cells from other independent studies (Fig. 4B-C). Consistent with these findings, the qRT-PCR results revealed that, compared with the other four genes, NSDHL was more strongly upregulated in T24^GemR^ cells (Fig. 4D). Furthermore, NSDHL mRNA levels increased progressively in T24 and UM-UC-3 cells with increasing GEM concentrations (Fig. 4E-F). Given that E2F1 is a canonical transcription factor, we hypothesized that it might transcriptionally activate NSDHL to regulate cholesterol homeostasis. Analyses of the GEPIA database and GSE13507 dataset revealed a strong positive correlation between E2F1 and NSDHL mRNA levels (Fig. 4G-H). Heatmap visualization of published transcriptomic data from E2F1-knockdown models further revealed a decrease in NSDHL mRNA levels upon E2F1 depletion (Fig. 4I). Subsequent qRT-PCR and Western blot analyses confirmed that the knockdown of E2F1 in T24 and UM-UC-3 cells led to a modest but statistically significant decrease in NSDHL mRNA levels and significantly reduced the protein levels of NSDHL, paralleling the downregulation of the positive control gene CCNE1 (Fig. 4J-L). To validate direct transcriptional regulation, we analyzed multiple E2F1 ChIP-seq datasets via IGV software, which identified a prominent E2F1 binding peak within the NSDHL promoter region (Fig. 4M). Additionally, the JASPAR database was employed to analyze predicted potential E2F1 binding sequences in the NSDHL promoter (Fig. 4N). Collectively, these findings prompted us to investigate whether E2F1 functions as a transcription factor for NSDHL. Initially, the NSDHL promoter region (-2000 to -1, transcriptional start site is 0) underwent amplification, followed by cloning and insertion into the pGL4.10 vector (Fig. 4O). Subsequent dual-luciferase reporter assays demonstrated that exogenous E2F1 markedly enhanced NSDHL promoter activity (Fig. 4P). However, after we mutated the predicted binding sequence to CCTATATTGCT, the enhancement effect of exogenous E2F1 on the activity of the mutant NSDHL promoter was significantly weakened (Fig. 4O-P). To ascertain direct E2F1 binding to the NSDHL promoter, ChIP-qPCR experiments were conducted. The results revealed that E2F1 enriched DNA fragments within the NSDHL promoter region (Fig. 4Q-R). Together, these data indicate that E2F1 is able to bind to the promoter region of NSDHL and enhance NSDHL transcription, demonstrating that E2F1 is a transcription factor for NSHDL.

### NSDHL is a functional downstream target of E2F1 that promotes GEM resistance in BLCA

**Fig. 5:**
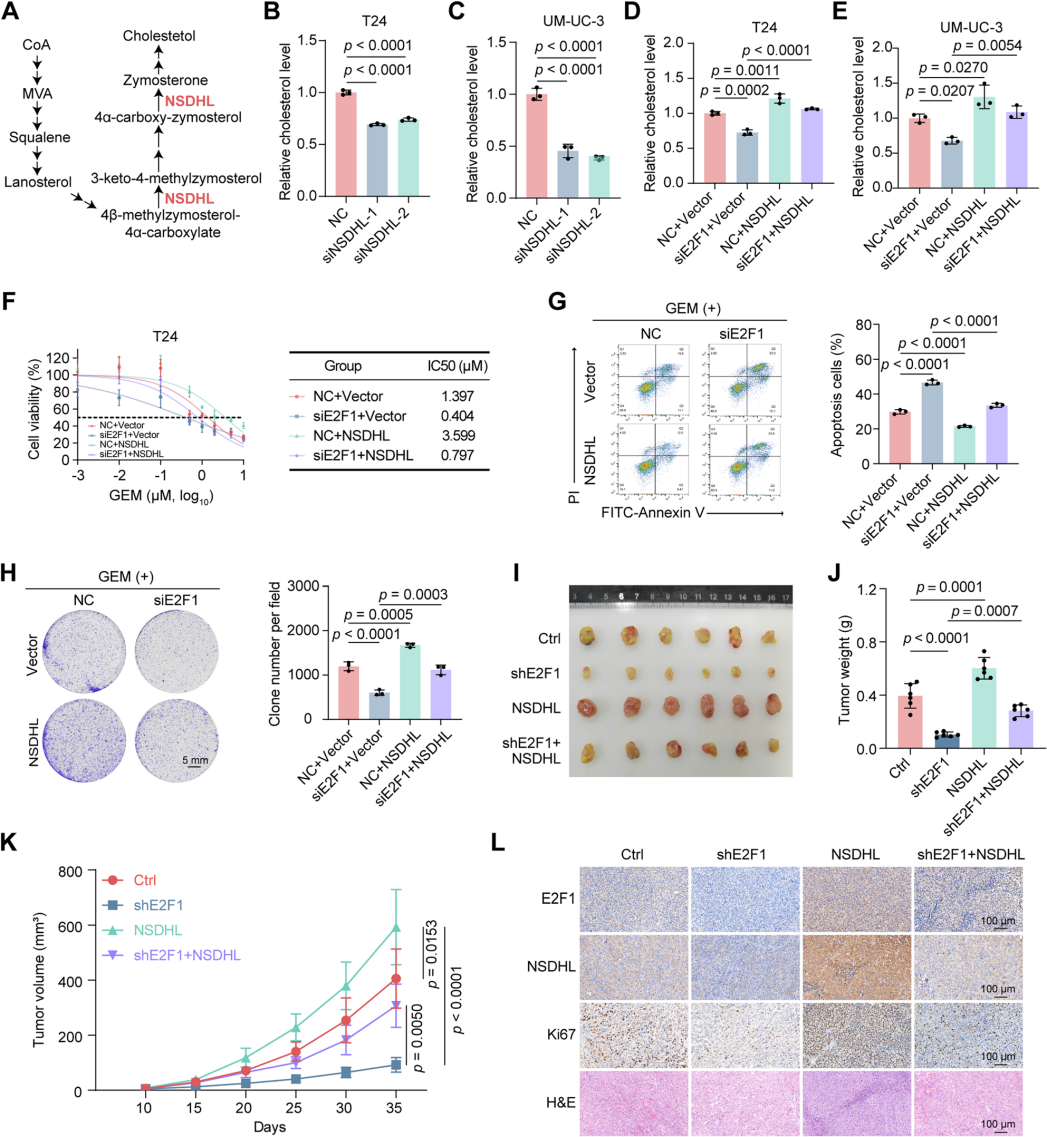
NSDHL is a functional downstream target of E2F1 that promotes GEM resistance in BLCA. **A** A brief schematic of the cholesterol biosynthesis process. **B**-**C** Relative cholesterol levels in T24 (B) and UM-UC-3 cells (**C**) after NSDHL knockdown (n = 3). **D**-**E** Relative cholesterol levels in T24 cells (**D**) and UM-UC-3 cells (**E**) in the indicated groups (n = 3). **F** Cell viability and IC50 values of T24 cells in the indicated groups after 48 hrs of treatment with different concentrations of GEM, as determined via the MTT assay (n = 5). **G** Apoptosis analysis of T24 cells in the indicated groups after 48 hrs of treatment with 1 μM GEM (n = 3). **H** Colony formation assay of T24 cells in the indicated groups after 48 hrs of treatment with 1 μM GEM (n = 3). **I** Gross view of subcutaneous xenograft tumors from the indicated groups (n = 6). **J** The tumor weights of the indicated groups at the endpoint (n = 6). **K** The tumor volume of the indicated group was measured on different days (n = 6). **L** Representative images of H&E and IHC staining of subcutaneous xenograft tumors from the indicated groups. The scale bar is 100 μm. Statistical significance was determined by one-way ANOVA with Dunnett’s multiple comparisons (**B**, **C**) and one-way ANOVA with Tukey’s multiple comparisons test (**D**, **E**, **G**, **H**, **J**, **K**). The data are shown as the means ± SDs

NSDHL is a key enzyme in cholesterol biosynthesis (Fig. 5A). Our previous findings established the critical role of cholesterol homeostasis in GEM resistance in BLCA. Thus, we hypothesized that NSDHL, a key enzyme in cholesterol biosynthesis, might contribute to GEM resistance. Analysis of the GEPIA database revealed significantly higher NSDHL expression in BLCA tissues than in normal tissues (Supplementary Fig. S3A). Consistent with these findings, evaluation of the GSE13507 dataset revealed elevated NSDHL levels in BLCA tissues compared with adjacent normal tissues, with progressive upregulation correlating with higher pathological grades (Supplementary Fig. S3B-C). The TCGA BLCA dataset further indicated that patients with high NSDHL expression exhibited reduced disease-free survival (Supplementary Fig. S3D). In addition, nonpapillary tumors, which are more aggressive histologically, presented higher NSDHL expression than papillary tumors (Supplementary Fig. S3E). Moreover, qRT-PCR analysis of the BLCA tissues from the ZNWU in-house dataset showed significantly elevated NSDHL expression in tumor tissues relative to matched adjacent normal tissues (Supplementary Fig. S3F). To validate the role of NSDHL in modulating cholesterol biosynthesis and GEM resistance, we knocked down NSDHL in T24 and UM-UC-3 cells, which significantly reduced intracellular cholesterol levels (Fig. 5B-C and Supplementary Fig. S3G-H) and enhanced GEM sensitivity (Supplementary Fig. S3I-N). Given that NSDHL is a downstream target of E2F1, we investigated whether NSDHL functionally mediates E2F1-driven cholesterol biosynthesis and chemoresistance. Notably, overexpression of NSDHL rescued the reduced cholesterol levels caused by E2F1 knockdown (Fig. 5D-E). Cell viability, apoptosis, and colony formation assays confirmed that NSDHL overexpression effectively counteracted the increased GEM sensitivity resulting from E2F1 depletion (Fig. 5F-H). These in vitro findings were also corroborated in vivo. A subcutaneous xenograft model demonstrated that NSDHL overexpression restored the tumor growth rate and weight under GEM treatment despite E2F1 knockdown (Fig. 5I-L). Briefly, we found that NSDHL is a direct downstream target of E2F1 that promotes GEM resistance in BLCA.

### NSDHL-dependent cholesterol accumulation enhances ABC transporter efflux and suppresses ferroptosis

We next explored the downstream functional consequences of NSDHL-dependent cholesterol accumulation in GEM-resistant BLCA cells, focusing on drug efflux and ferroptosis. First, we employed the EFLUXX-ID Green Multidrug Resistance Assay to measure the efflux activity of major ABC transporters (MDR1, MRP, and BCRP), which are responsible for drug efflux. We found that intracellular MFI was significantly lower in T24^GemR^ cells than in parental T24 cells, indicating substantially higher ABC transporter-mediated efflux activity in the resistant cells (Supplementary Fig. S4A-B). Knockdown of NSDHL in both T24 and T24^GemR^ cells increased MFI to varying degrees, suggesting that NSDHL depletion reduces ABC transporter-mediated efflux (Supplementary Fig. S4B). Moreover, in T24^GemR^ cells, inhibition of MRP caused the most pronounced rise in MFI, indicating that MRP represents the dominant transporter contributing to drug efflux in this setting (Supplementary Fig. S4C).

We next investigated whether NSDHL-dependent cholesterol accumulation affects ferroptosis. As shown in Supplementary Fig. S4D, the intracellular ferrous iron (Fe^2+^) level was significantly lower in T24^GemR^ cells than in parental T24 cells. Notably, NSDHL knockdown markedly increased Fe^2+^ levels in both T24 and T24^GemR^ cells. We further assessed lipid ROS accumulation using the BODIPY-C11 fluorescent probe, as lipid peroxidation is a key feature of ferroptosis. T24^GemR^ cells were partially resistant to Erastin-induced lipid peroxidation compared with parental cells. In contrast, knockdown of NSDHL significantly exacerbated Erastin-induced lipid ROS accumulation in both T24 and T24^GemR^ cells (Supplementary Fig. S4E). Together, these findings indicate that GEM-resistant cells display enhanced ferroptosis resistance, and that NSDHL depletion re-sensitizes both parental and resistant cells to ferroptosis.

### 2,3-Oxidosqualene is the sterol intermediate responsible for NSDHL-mediated GEM resistance

Given that NSDHL-dependent cholesterol accumulation contributes to GEM resistance, we next sought to investigate which specific sterol intermediates in the cholesterol biosynthetic pathway are responsible for promoting this resistance. Due to technical limitations in detecting unstable and low-abundance sterol intermediates, comprehensive profiling of the entire cholesterol biosynthesis pathway is currently challenging. Thus, based on clinical relevance, analytical feasibility, and scientific value, we selected 12 representative sterol intermediates for targeted cholesterol metabolomics. We observed that total cholesteryl ester content was significantly higher in T24^GemR^ cells than in parental T24 cells (Supplementary Fig. S4F). Among the profiled intermediates, five sterols (Lanosterol, 2,3-Oxidosqualene, Dihydrolanosterol, Sitosterol, and Campesterol) were elevated in T24^GemR^ cells, and each was reduced upon NSDHL knockdown (Supplementary Fig. S4F). We therefore selected these five sterols for further functional screening. In GEM cytotoxicity assays, only exogenous 2,3-Oxidosqualene significantly rescued cells from GEM-induced death (Supplementary Fig. S4G). IC50 analyses further showed that 2,3-Oxidosqualene supplementation reversed the increased GEM sensitivity caused by NSDHL depletion (Supplementary Fig. S4H). Collectively, these data identify 2,3-Oxidosqualene as a sterol species functionally responsible for driving GEM resistance downstream of NSDHL-dependent cholesterol biosynthesis.

### USP43 interacts with E2F1

**Fig. 6:**
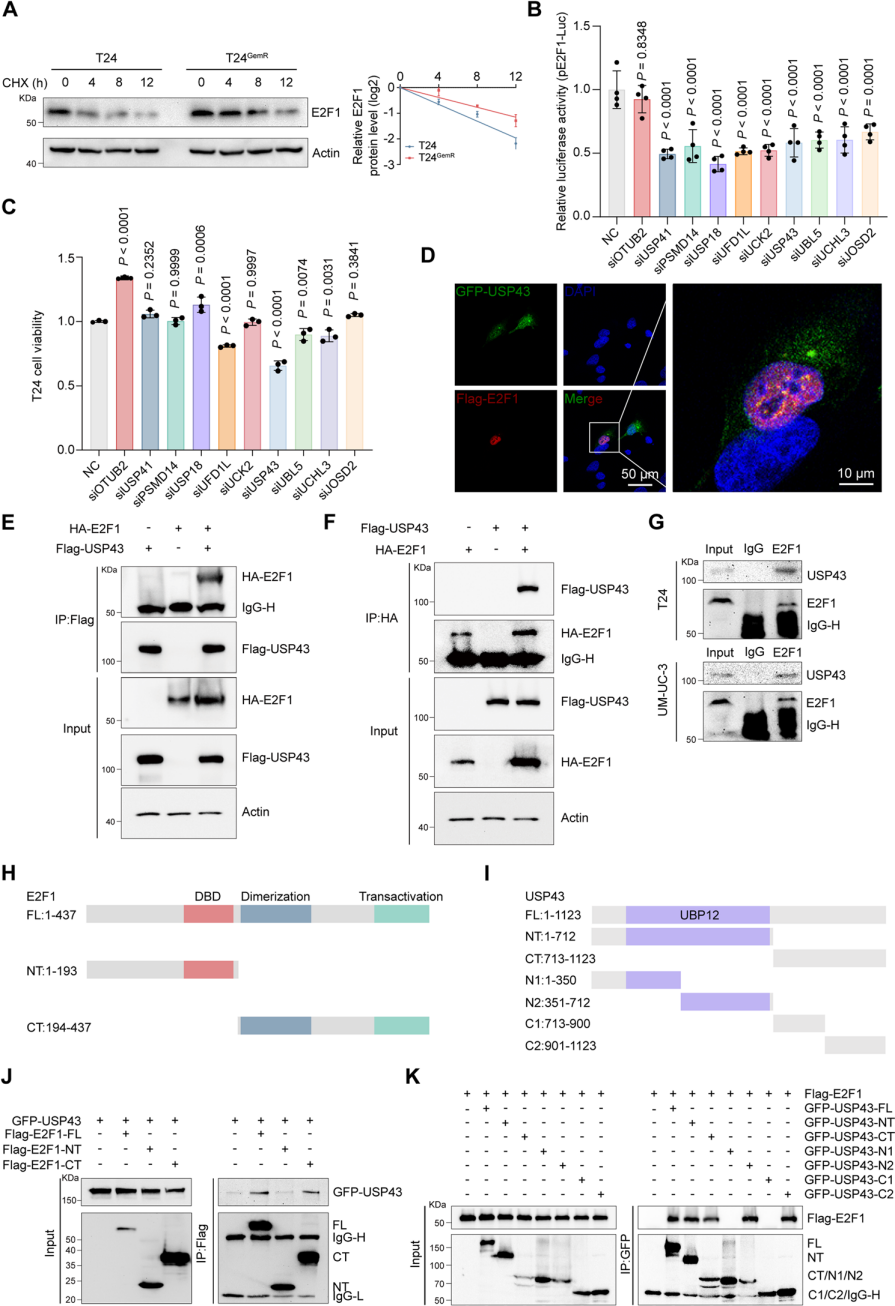
USP43 interacts with E2F1. **A** Representative Western blot images (left panel) and statistical results (right panel) of the E2F1 half-life in T24 and T24^GemR^ cells. All the cells were treated with 50 μg/mL CHX for the indicated times (n = 3). **B** Ten siRNAs of DUBs highly expressed in BLCA from a DUB siRNA library and negative control were transfected into 293T cells. Dual-luciferase reporter assay showing the promoter activity of E2F1 target genes after knockdown of various DUBs (n = 4). **C** Viability of T24 cells with various DUB knockdowns after treatment with 1 μM GEM for 48 hrs, as detected via the MTT assay (n = 3). **D** Confocal immunofluorescence images showing that USP43 and E2F1 were colocalized in the nuclei of UM-UC3 cells. The scale bar is 50 μm. **E**-**F** Co-IP assays showing the interaction between exogenous USP43 and E2F1 in 293T cells. Cells were co-transfected with Flag-USP43 and HA-E2F1, followed by Flag-IP (**E**) or HA-IP (**F**). Input fractions include Actin loading controls. **G** Co-IP revealed that endogenous USP43 interacted with E2F1 in T24 and UM-UC-3 cells. **H**-**I** Schematic illustrations of E2F1 (**H**) and USP43 (**I**) truncations. **J** GFP-USP43 and full-length or truncated mutants of Flag-E2F1 were transfected into 293T cells, and a Co-IP assay was used to detect the interactions. **K** Flag-E2F1 and full-length or truncated mutants of GFP-USP43 were transfected into 293T cells, and a Co-IP assay was used to detect the interactions. Statistical significance was determined by one-way ANOVA with Dunnett’s multiple comparisons test (**B**, **C**). The data are shown as the means ± SDs

Our comparative analysis demonstrated that T24^GemR^ cells displayed significantly higher E2F1 expression levels than T24 cells at both mRNA and protein levels. Moreover, GEM treatment induced marked upregulation of E2F1 expression at mRNA and protein levels (Fig. 1E-I). Previous studies have reported that E2F1 protein stability is frequently modulated by post-translational modifications (PTMs) [29–31]. Our cycloheximide (CHX) assay demonstrated greater protein stability of E2F1 in T24^GemR^ cells than in T24 cells (Fig. 6A), indicating that PTMs contribute to E2F1 upregulation during GEM resistance. Generally, the balance between ubiquitination and deubiquitination critically regulates substrate protein stability [32]. Therefore, we subsequently aimed to identify deubiquitinases (DUBs) that play a key role in GEM-induced E2F1 upregulation. First, we selected ten deubiquitinases highly expressed in BLCA from a deubiquitinase library (Supplementary Fig. S5). We subsequently performed a screen using a dual-luciferase reporter system reflecting E2F1 transcriptional activity. The results revealed that the knockdown of OTUB2 did not affect E2F1 transcriptional activity, whereas the knockdown of the remaining nine candidate DUBs caused various degrees of reduction in E2F1 transcriptional activity (Fig. 6B). We subsequently performed a second round of screening on the basis of the impact of GEM treatment on sensitivity in BLCA patients. The results revealed that only the knockdown of UFD1L, USP43, UBL5, or UCHL3 increased GEM sensitivity, with USP43 knockdown resulting in the most significant effect (Fig. 6C). These results indicate that USP43 may serve as a pivotal regulator of E2F1 PTMs. Immunofluorescence experiments revealed that USP43 co-localized with E2F1 in the nucleus of UM-UC-3 cells (Fig. 6D). To validate USP43-E2F1 interaction, a Co-IP assay was conducted. The results revealed a prominent interaction between Flag-USP43 and HA-E2F1, with both being exogenously expressed in 293T cells (Fig. 6E-F). USP43 overexpression also clearly increases HA-E2F1 levels in the input, consistent with a stabilizing effect (Fig. 6E). Additionally, endogenous USP43 also interacted with E2F1 in T24 and UM-UC-3 cells (Fig. 6G). A set of truncated mutant plasmids was constructed for subsequent co-IP experiments to delve deeper into the specific structural domains involved in the binding of USP43 and E2F1. As shown in Fig. 6H-K, USP43 bound to the C-terminal domain of E2F1, whereas E2F1 interacted with both the USP43-N2 and USP43-C2 regions of USP43. These findings conclusively indicate a physical interaction between USP43 and E2F1.

### USP43 deubiquitinates and stabilizes E2F1

**Fig. 7:**
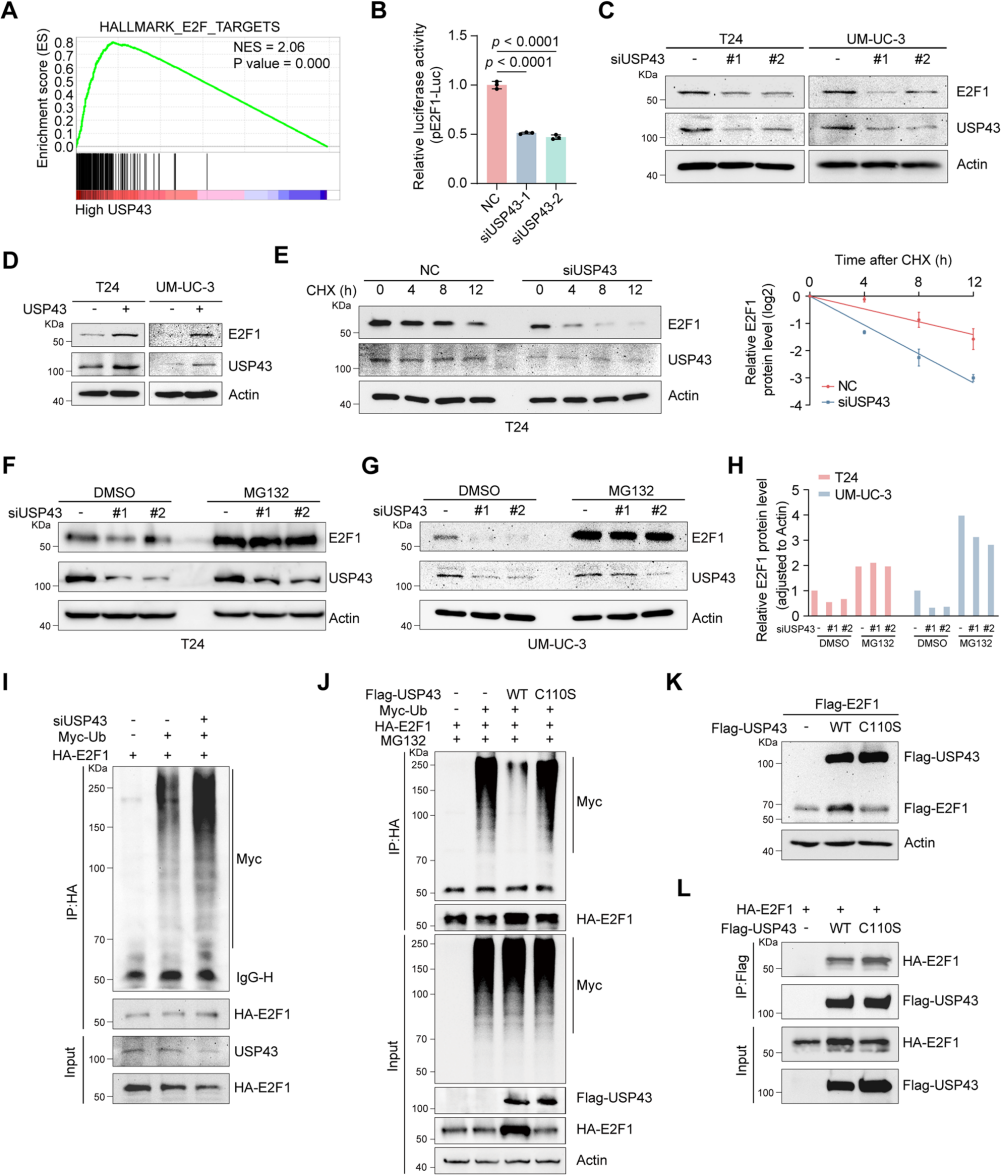
USP43 deubiquitinates and stabilizes E2F1. **A** GSEA of the TCGA database revealed that USP43 is positively related to the E2F target pathway. **B** Dual-luciferase reporter assay showing the promoter activity of E2F1 target genes after USP43 knockdown (n = 3). **C**-**D** Western blot analysis of the effects of USP43 knockdown (**C**) or overexpression (**D**) on the E2F1 protein in T24 and UM-UC-3 cells. **E** Representative Western blot images (left panel) and statistical results (right panel) of the effect of USP43 knockdown on the E2F1 half-life in T24 cells. T24 cells were transfected with siNC or siUSP43 for 48 hrs and then incubated with 50 μg/mL CHX for the indicated times (n = 3). **F**-**H** T24 (**F**) and UM-UC-3 (**G**) cells were transfected with siNC or siUSP43 for 48 hrs and then treated with DMSO or 10 μM MG132 for 6 hrs, followed by harvesting for Western blot analysis. The statistical results revealed the protein level of E2F1, with actin serving as a loading control (**H**). **I** 293T cells were transfected with the siRNAs and plasmids as indicated for 48 hrs and then treated with 10 μM MG132 for 6 hrs. Ubiquitination experiments were performed to explore the effect of USP43 on E2F1 ubiquitination. **J** 293T cells transfected with HA-E2F1, Myc-Ubiquitin or Flag-USP43 (wild-type or C110S) were treated with 10 μM MG132 for 6 hrs before collection, after which ubiquitination experiments were performed to analyze the polyubiquitination of E2F1. Input fractions include Actin loading controls. **K** The described plasmids were transfected into 293T cells for 48 hrs. A western blot analysis was used to analyze the protein level of E2F1. **L** 293T cells were transfected with HA-E2F1 and Flag-USP43 (wild-type or C110S). A co-IP assay was used to detect the interaction of wild-type USP43 and the enzyme-inactivated mutant (C110S) with E2F1. Statistical significance was determined by one-way ANOVA with Dunnett’s multiple comparisons test (B). The data are shown as the means ± SDs

After demonstrating that USP43 interacts with E2F1, we wanted to further clarify that USP43 functions as a deubiquitinase (DUB) for E2F1. GSEA of USP43 via the TCGA database revealed that USP43 can be enriched in the E2F target pathway (Fig. 7A). A dual-luciferase assay confirmed that USP43 knockdown was able to reduce the transcriptional activity of E2F1 on downstream target genes (Fig. 7B). USP43 knockdown in T24 cells resulted in a modest increase in E2F1 mRNA levels, while in UM-UC-3 cells it caused a slight reduction (Supplementary Fig. S6A-B). However, USP43 knockdown dramatically decreased the protein level of E2F1 (Fig. 7C). Conversely, USP43 overexpression increased the protein level of E2F1 (Fig. 7D). Moreover, USP43 increased the protein level of E2F1 in a dose-dependent manner (Supplementary Fig. S6C). Next, we knocked down USP43 in T24 cells and executed a CHX chase assay to assess whether USP43 impacts E2F1 protein stability. Our results revealed that the half-life of the E2F1 protein was significantly shortened after USP43 knockdown (Fig. 7E). In addition, the proteasome inhibitor MG132 blocked USP43’s effect on E2F1 (Fig. 7F-H). These findings indicate that USP43 maintains the stability of E2F1 via the ubiquitin-proteasome pathway. Subsequently, USP43’s effect on E2F1 polyubiquitination levels was assessed. Consistent with expectations, USP43 knockdown resulted in a significant increase in E2F1 polyubiquitination levels (Fig. 7I). In a previous study, we constructed a USP43 DUB inactivating mutant through substitution of the cysteine at position 110 with serine (C110S) [26]. The overexpression of USP43 led to a marked reduction in E2F1 polyubiquitination levels, whereas the C110S mutant exhibited comparable E2F1 binding affinity to wild-type USP43 yet demonstrated reduced efficacy in decreasing E2F1 polyubiquitination levels and elevating E2F1 protein content (Fig. 7J-L). Similarly, wild-type USP43 was able to significantly prolong the half-life of E2F1, whereas the C110S mutant was not (Supplementary Fig. S6D). Collectively, these results suggest that USP43 is a DUB of E2F1 and can stabilize E2F1 through the ubiquitin-proteasome pathway.

### USP43 is essential for GEM-induced E2F1 upregulation and is a novel chemoresistance target

**Fig. 8:**
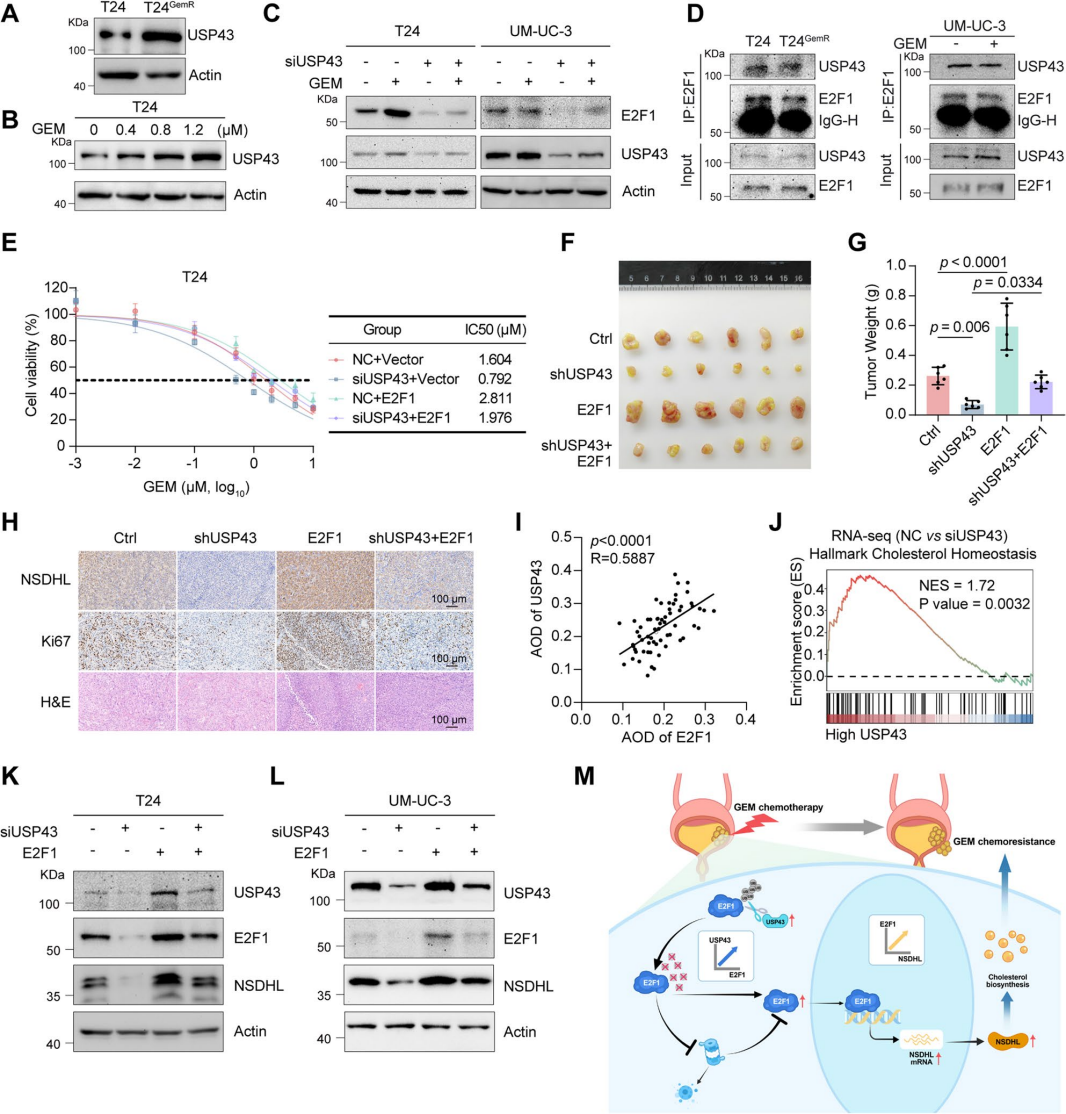
USP43 is essential for GEM-induced E2F1 upregulation and is a novel chemoresistance target. **A** Western blot assays showing the expression of USP43 in T24 and T24^GemR^ cells. **B** T24 cells were treated with different concentrations of GEM for 48 hrs, and UPS43 was detected by subsequent immunoblot analysis. **C** Western blots showing the expression of USP43 and E2F1 in T24 and UM-UC-3 cells after USP43 knockdown and treatment with or without GEM for 48 hrs. **D** Co-IP assays revealed that endogenous USP43 interacts with E2F1 in T24 and T24^GemR^ cells (left diagram) and in UM-UC-3 cells with or without GEM treatment (right diagram). **E** Cell viability and IC50 values of T24 cells in the indicated groups after 48 hrs of treatment with different concentrations of GEM, as measured by the MTT assay (n = 5). **F** Gross view of subcutaneous xenograft tumors from the indicated groups (n = 6). **G** The tumor weights of the indicated groups at the endpoint (n = 6). **H** Representative images of H&E and IHC staining of subcutaneous xenograft tumors from the indicated groups. **I** The correlation between USP43 and E2F1 protein expression was evaluated via IHC staining in overlapping patients from the HBlaU079Su01 and HBlaU108Su01. **J** GSEA of RNA-seq data from USP43-knockdown T24 cells suggested that USP43 is involved in the cholesterol homeostasis pathway. **K**-**L** Western blots showing the protein expression of USP43, E2F1 and NSDHL in T24 (**K**) and UM-UC-3 (**L**) cells after USP43 knockdown and E2F1 overexpression. **M** Mechanism diagram of this study. Statistical significance was determined by one-way ANOVA with Tukey’s multiple comparisons test (**G**) and Pearson’s correlation (**I**). The data are shown as the means ± SDs

Because USP43 can regulate the stability of E2F1, we wanted to investigate the role of USP43 in the process of GEM resistance in BLCA. First, we examined the expression of USP43 in T24 and T24^GemR^ cells and found that USP43 was expressed at a relatively high level in the T24^GemR^ cell line (Fig. 8A). We subsequently stimulated the cells with different concentrations of GEM and found that GEM increased the protein level of USP43 in a dose-dependent manner (Fig. 8B). Next, we wanted to clarify whether knocking down USP43 could affect the E2F1 upregulation induced by GEM. The experimental results revealed that GEM was able to induce the upregulation of E2F1 expression, but the GEM-induced upregulation of E2F1 was blocked after USP43 was knocked down, suggesting that USP43 is necessary for GEM-induced E2F1 upregulation (Fig. 8C). We then proceeded to explore whether GEM stimulation affects the binding of both USP43 and E2F1. The results of endogenous IP demonstrated that there was no significant difference in the degree of binding between USP43 and E2F1 in T24 and T24^GemR^ cells. Similarly, the addition or absence of GEM did not affect the binding of the two proteins in UM-UC-3 cells (Fig. 8D). Consequently, GEM does not appear to increase USP43-E2F1 binding per se, but rather acts upstream by elevating USP43 levels, which then contributes to sustaining E2F1 protein stability. Given the critical role of USP43 in mediating the GEM-induced upregulation of E2F1 expression, this DUB is postulated to regulate GEM resistance in BLCA. Consistent with our hypothesis, the results of the cell viability, cell apoptosis and colony formation assays confirmed that USP43 knockdown enhanced the sensitivity of BLCA cells to GEM (Supplementary Fig. S7A-F). Given the established role of USP43 in stabilizing E2F1, we investigated whether USP43-mediated GEM resistance in BLCA depends on E2F1. In vitro cell viability assays, cell apoptosis assays and colony formation assays demonstrated that E2F1 overexpression significantly counteracted the enhanced GEM sensitivity resulting from USP43 deficiency (Fig. 8E and Supplementary Fig. S7G-H). This rescue effect was further validated in vivo via the use of subcutaneous xenograft models, in which E2F1 overexpression reversed the USP43 silencing-induced suppression of tumor growth (Fig. 8F-H and Supplementary Fig. S7I). These findings collectively confirm that USP43 promotes GEM resistance primarily through E2F1.

Building upon our previous findings, we propose a coherent biological mechanism whereby USP43 stabilizes E2F1 through its DUB activity, enabling E2F1-mediated transcriptional activation of NSDHL to potentiate cholesterol biosynthesis and GEM resistance. A comprehensive series of experiments further validated this axis: tissue microarray analysis of BLCA samples revealed that USP43 and E2F1 protein levels were positively correlated (Fig. 8I). GSEA of RNA-seq data from USP43-depleted T24 cells demonstrated marked suppression of cholesterol homeostasis pathways (Fig. 8J). TCGA analysis via GEPIA demonstrated significant co-expression between USP43 and NSDHL transcripts (Supplementary Fig. S7J). qRT-PCR confirmed that USP43 knockdown reduced NSDHL mRNA levels (Supplementary Fig. S7K). Western blot analysis confirmed that E2F1 overexpression rescued NSDHL protein reduction in USP43-silenced cells (Fig. 8K-L). Finally, qRT-PCR analysis confirmed significantly higher USP43 expression in tumor tissues compared to matched adjacent normal tissues from the ZNWU in-house dataset (Supplementary Fig. S7L). These data establish the USP43/E2F1/NSDHL axis as a critical regulator of cholesterol biosynthesis-driven GEM resistance in BLCA. Our findings highlight USP43 as both a novel therapeutic target and a predictive biomarker for overcoming chemoresistance in BLCA treatment (Fig. 8M).

## Discussion

Gemcitabine (GEM), a deoxycytidine analog that targets S-phase progression, serves as a crucial chemotherapeutic agent for various solid tumors. The prodrug exerts its antineoplastic effects following its intracellular phosphorylation to its active triphosphate metabolite (dFdCTP), which can be incorporated into DNA as a false nucleoside, thereby disrupting DNA replication processes [33]. Although numerous novel perspectives and therapeutic approaches for cancer have emerged [34], GEM is still a pivotal chemotherapy used as a first-line treatment for BLCA currently. GEM is employed across therapeutics, including monotherapies and intravesical treatments immediately following TURBT for NMIBC, combination regimens for MIBC, and neoadjuvant chemotherapy, and even serves as a radiosensitizer [2, 35–37]. However, the therapeutic efficacy of GEM in BLCA remains suboptimal. For patients treated with cisplatin and GEM, clinical drug resistance is a persistent and unavoidable barrier to therapeutic success. Hence, uncovering drivers of GEM resistance and identifying novel strategies to increase their therapeutic efficacy are key priorities. In this study, to uncover the molecular mechanisms underlying GEM resistance in BLCA, we systematically analyzed publicly available sequencing data and established GEM-resistant BLCA cell lines. Through these approaches, we identified E2F1 as a key driver of GEM resistance in BLCA. Our team previously demonstrated that E2F1 plays crucial roles in BLCA proliferation and metastasis [28]. These findings significantly expand our understanding of the functional repertoire of E2F1 in BLCA pathogenesis.

A shared hallmark of various cancers is the reprogramming of lipid metabolism. Lipids serve as indispensable biomolecules in cancer cells, functioning as structural elements of cellular membranes, signaling mediators and energy reservoirs, driving the progression and enhancing the proliferation of tumors [38]. Cholesterol, an important lipid component, has a variety of cellular biological functions, including being a component of biological membranes, a precursor of bile acids and steroid hormones, and serves as a signaling mediator. Emerging research highlights the importance of dysregulated cholesterol metabolism in tumorigenesis. For example, dysregulated cholesterol homeostasis generates ferroptosis-resistant cancer cells, leading to increased tumor growth and metastasis [39]. ANO1 promotes cancer metastasis in esophageal squamous cell carcinoma (ESCC) by inhibiting CYP27A1-LXR signaling, which leads to increased cholesterol levels and reprogramming of the tumor microenvironment [40]. Nevertheless, the function of cholesterol in GEM resistance in BLCA remains unclear. This study demonstrated that GEM-resistant cells presented elevated cholesterol levels and established the functional contribution of cholesterol to GEM resistance in BLCA. However, our current work did not delineate the specific roles of cholesterol intermediates or derivatives in mediating drug resistance, which represents a limitation of this investigation. We intend to address this knowledge gap systematically in future studies through comprehensive metabolic profiling and functional validation.

E2F1, a classical cell cycle regulator, has been shown to be involved in the control of lipid metabolism. In fatty liver disease, E2F1 regulates lipid biosynthesis, thereby promoting hepatic pathology progression [16]. Furthermore, E2F1 contributes to lipogenesis in progenitor cells and medulloblastoma [41]. However, the role of E2F1 in chemoresistance and lipid metabolism in BLCA has not yet been fully revealed. In this study, we found that E2F1 is highly expressed in BLCA and that E2F1 activity is induced during chemotherapy with GEM in both in vitro and in vivo models, as evidenced by the increased mRNA and protein levels of E2F1. Moreover, E2F1 knockdown enhanced the sensitivity of BLCA cells to GEM. By analyzing several RNA-seq datasets through GSEA, we revealed that E2F1 may enhance GEM resistance in BLCA through lipid metabolism and cholesterol homeostasis pathways. Furthermore, exogenous cholesterol supplementation reversed the increase in GEM sensitivity resulting from E2F1 knockdown in BLCA cells, which supported our previous conclusion. E2F1 has dual effects on tumor development, with its downstream target encompassing both oncogenic drivers and tumor suppressors [42]. Here, we identified NSDHL, a rate-limiting enzyme in the cholesterol biosynthesis pathway, as the functional downstream target of E2F1 that promotes GEM resistance in BLCA. Previously, only a few studies have explored the role of NSDHL in tumors. For the first time, we revealed that the knockdown of NSDHL lowered cholesterol levels and enhanced GEM sensitivity in BLCA. On the other hand, overexpression of NSDHL partially counteracted the increased sensitivity to GEM caused by E2F1 knockdown.

E2F1 protein stability is tightly controlled through the ubiquitin-proteasome pathway. In our research, we used a siRNA library targeting DUBs to identify those that enhance E2F1 transcriptional activity and contribute to GEM resistance in BLCA. We discovered that USP43, a crucial DUB, plays a significant role in enhancing GEM resistance and E2F1 transcriptional activity. USP43 interacts with E2F1, removing its ubiquitin tags and thereby preventing its degradation through the ubiquitin-proteasome pathway. Notably, USP43-mediated deubiquitination of E2F1 is likely to occur in the nucleus and that this is consistent with previously described nuclear deubiquitination events [43–45]. This process leads to an accumulation of E2F1, which activates the transcription of NSDHL, a gene involved in cholesterol synthesis. This action forms part of the USP43/E2F1/NSDHL axis, which we found to be essential in promoting resistance to GEM in BLCA. Disruption of this axis results in altered cholesterol homeostasis and increased level of E2F1 protein, which together drive the GEM resistance. These findings highlight the role of abnormal USP43-mediated expression of E2F1 in disrupting cholesterol homeostasis and inducing GEM resistance in BLCA (Fig. 8M). Given the promising potential of DUBs in targeted drug development [46], future research should aim at developing specific inhibitors of USP43 to move these discoveries towards clinical applications.

In our earlier study, USP43 was shown to stabilize c-Myc to promote glycolysis and metastasis [26], while E2F1 was studied in the context of DLGAP5/USP11-mediated stabilization driving proliferation and metastasis [28]. In contrast, this study focuses on GEM resistance and identifies a USP43-E2F1-NSDHL axis that regulates cholesterol biosynthesis. Here, USP43 was found to be elevated in GEM-resistant cells and overexpressed in BLCA tissues, and its modulation produced clear effects on GEM sensitivity in our functional assays (Fig. 8). These findings led us to focus on USP43 as the major deubiquitinase regulating E2F1 stability in GEM resistance in the present study. E2F1 stabilized by USP43 directly activates NSDHL transcription, leading to increased cholesterol accumulation and GEM resistance. Thus, our data extend the functional landscape of USP43 and E2F1 from metastasis and glycolytic reprogramming to cholesterol-mediated chemoresistance, and introduce NSDHL-dependent cholesterol metabolism as a downstream pathway of USP43/E2F1 that has not previously been linked to GEM resistance in BLCA.

However, our present study also has several limitations. We have established a correlation between elevated cholesterol levels and GEM resistance in BLCA, yet the underlying mechanisms remain incompletely understood. In addition, other processes associated with cholesterol homeostasis, including cholesterol uptake, transport, and elimination, have not been investigated.

## Conclusions

In conclusion, our study identified USP43 as a critical regulator of cholesterol homeostasis and E2F1 activity in BLCA. Consequently, USP43 is expected to be a viable therapeutic target to disrupt the USP43/E2F1/NSDHL axis and enhance GEM sensitivity in BLCA.

## Supplementary Information

Supplementary Material 1.

## Data Availability

Our RNA-seq data have been uploaded to the Gene Expression Omnibus (GEO) database with the accession number GSE299182. Reviewer access: Go to https://www.ncbi.nlm.nih.gov/geo/query/acc.cgi? acc=GSE299182, enter token gzgfeomybraxvqd into the box. The publicly available data for differential gene expression analysis and survival analysis were obtained from the online website GEPIA (http://gepia.cancer-pku.cn/index.html). The publicly available TCGA-BLCA data (the data included 408 tumors and 19 normal samples) were obtained from the GDC Data Portal website (https://portal.gdc.cancer.gov/). Data from the GSE190636, GSE77883, GSE121258 and GSE13507 datasets are publicly available in the GEO database and were used in this study. The publicly available E2F1 ChIP-seq datasets were retrieved from the GEO database under accession numbers GSE62425, GSE77448, GSE67809, GSE49832, GSE99171 and GSE94958. The remaining data are available within the article, Supplementary Information or Source Data file.

## Abbreviations

BLCA: Bladder cancer
ChIP: Chromatin immunoprecipitation
CHX: Cycloheximide
DMSO: Dimethyl sulfoxide
DUB: Deubiquitinase
DUBs: Deubiquitinases
ESCC: Esophageal squamous cell carcinoma
GC: Gemcitabine + Cisplatin
GEM: Gemcitabine
GSEA: Gene set enrichment analysis
H&E: Hematoxylin and eosin
IHC: Immunohistochemistry
MFI: Mean fluorescence intensity
MIBC: Muscle-invasive bladder cancer
MTT: Methyl thiazolyl tetrazolium
NMIBC: Non-muscle-invasive bladder cancer
PI: Propidium iodide
PTMs: Post-translational modifications
qPCR: Quantitative PCR
qRT-PCR: RNA extraction and quantitative reverse transcription PCR
RNA-seq: RNA sequencing
SDs: Standard deviations
siRNAs: Small interfering RNAs
STR: Short tandem repeat
TURBT: Transurethral resection of bladder tumor
USP: Ubiquitin-specific protease
ZNWU: Zhongnan Hospital of Wuhan University
