# Supplementary material for: USP43 promotes gemcitabine resistance by regulating cholesterol homeostasis through E2F1 stabilization in bladder cancer
Source: J Exp Clin Cancer Res. 2025 Dec 23;45:23. doi: 10.1186/s13046-025-03621-2 (PMC12838501; doi:10.1186/s13046-025-03621-2)
Supplement: Supplementary file 1 — Supplementary Material 1. [file 13046_2025_3621_MOESM1_ESM.pdf]

Supplementary Information

USP43 promotes gemcitabine resistance by regulating cholesterol homeostasis through E2F1 stabilization in bladder cancer

Supplementary Figures S1-S7: Pages 2-13

Supplementary Tables S1-S2: Pages 14-15

Supplementary Figures S1-S7

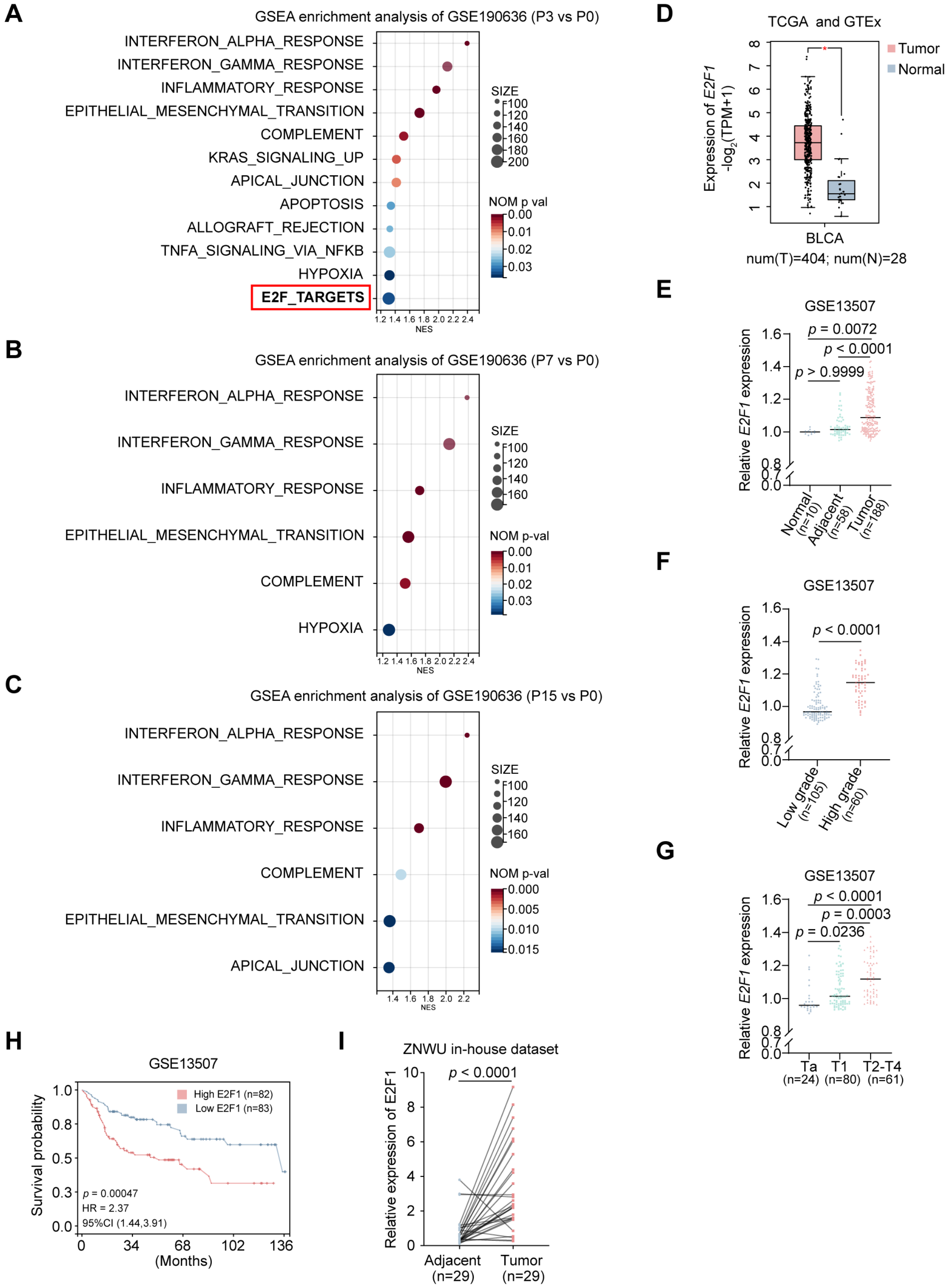

Supplementary Figure S1. E2F1 is induced by gemcitabine and is highly expressed in BLCA.

(A-C) GSEA enrichment analysis of GSE190636 at each time point: (A) P3 vs P0. (B) P7 vs P0. (C) P15 vs P0. **(D)** Comparative analysis of E2F1 expression levels between BLCA and normal tissues via the GEPIA database (*http://gepia.cancer-pku.cn/index.html*). $|Log_2FC|$ cutoff: 0.5, p value cutoff: 0.01. **(E)** mRNA levels of E2F1 in normal tissues (n = 10), adjacent tissues (n = 58) and BLCA (n = 188) in the GSE13507 (RNA-seq data). **(F)** The mRNA level of E2F1 in low-grade BLCA (n = 105) and high-grade BLCA (n = 60) in the GSE13507. **(G)** Relationships between E2F1 expression and BLCA T stage. Data were obtained from GSE13507. **(H)** Kaplan-Meier analysis was conducted to investigate the relationship between the E2F1 expression level and BLCA prognosis. The patients were divided into high-E2F1 and low-E2F1 mRNA expression groups according to the median E2F1 expression in the GSE13507 dataset. **(I)** mRNA expression levels of E2F1 in BLCA tissues (n = 29) and corresponding adjacent normal tissues (n = 29) from the ZNWU in-house dataset. Statistical significance was determined by Kruskal-Wallis test followed by Dunn's multiple comparisons test (E, G), Mann-Whitney test (F), the log-rank test of Kaplan-Meier analysis (H), and two-tailed paired Student's t test (I). The data are shown as the means ± SDs. * $p < 0.05$.

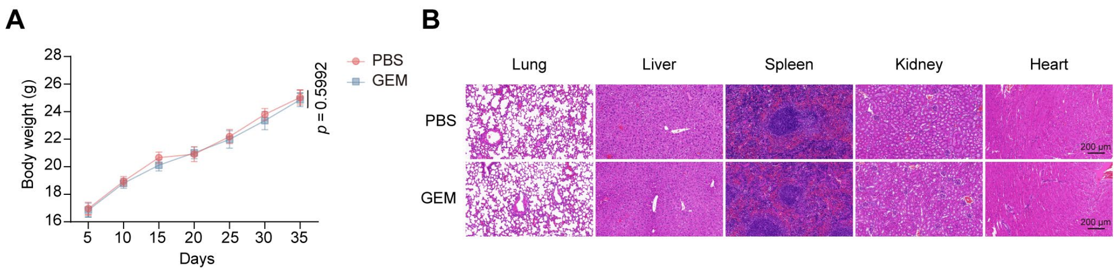

Supplementary Figure S2. GEM regimen shows no overt systemic toxicity in nude mice.

(A) Body-weight curves of nude mice treated with PBS or GEM (n = 5). **(B)** Representative H&E staining of major organs (lung, liver, spleen, kidney and heart) from PBS- and GEM-treated mice at endpoint, showing no overt pathological lesions. Statistical significance was determined by two-tailed paired Student's t test (A). The data are shown as the means ± SDs.

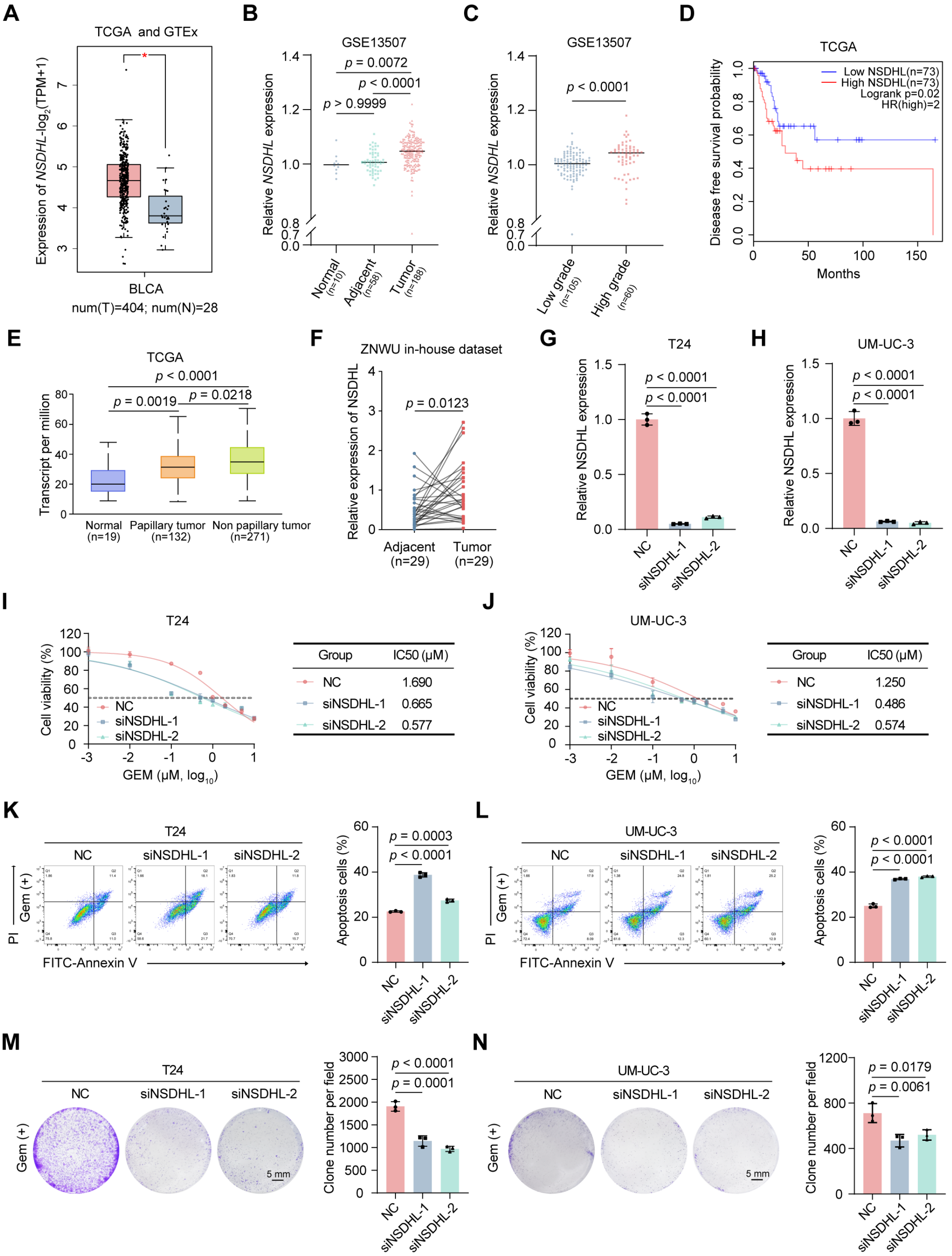

Supplementary Figure S3. NSDHL is highly expressed in BLCA and promotes gemcitabine resistance.

(A) Comparative analysis of NSDHL expression levels between BLCA and normal tissues via the GEPIA database (*http://gepia.cancer-pku.cn/index.html*). $|Log_2FC|$ cutoff: 0.5, p value cutoff: 0.01. **(B)** mRNA expression levels of NSDHL in normal tissues (n = 10), adjacent tissues (n = 58) and BLCA (n = 188) in the GSE13507 (RNA-

seq data). **(C)** The mRNA expression level of NSDHL in low-grade BLCA (n = 105) and high-grade BLCA (n = 60) samples in the GSE13507. **(D)** Kaplan-Meier analysis was performed to explore the relationship between the NSDHL expression level and BLCA prognosis. The patients were divided into high-E2F1 and low-E2F1 mRNA expression groups according to the median NSDHL expression in the TCGA. **(E)** mRNA expression levels of NSDHL in normal tissues (n = 19), papillary bladder tumors (n = 132) and nonpapillary bladder tumors (n = 237) in the TCGA. **(F)** mRNA expression levels of NSDHL in BLCA tissues (n = 29) and corresponding adjacent normal tissues (n = 29) from the ZNWU in-house dataset. **(G-H)** Quantification of NSDHL mRNA expression levels after NSDHL knockdown in T24 (F) and UM-UC-3 (G) cells via qRT-PCR. Apoptosis analysis of T24 cells in the indicated groups after 48 hrs of treatment with 1 μM GEM (n = 3). **(I-J)** Cell viability assay results showing the viability and IC50 values of T24 (H) and UM-UC-3 (I) cells with NSDHL knockdown after 48 hrs of exposure to various concentrations of GEM (n = 5). **(K-L)** Representative images (left panel) and statistical analysis (right panel) of apoptosis assays in T24 (J) and UM-UC3 (K) cells with NSDHL knockdown, followed by 1 μM GEM treatment for 48 hrs (n = 3). **(M-N)** Representative images (left panel) and statistical analysis (right panel) of colony formation assays in T24 cells (L) or UM-UC3 cells (M) with NSDHL knockdown, followed by 1 μM GEM treatment (n = 3). Statistical significance was determined by Kruskal-Wallis test followed by Dunn's multiple comparisons test (B), Mann-Whitney test (C), two-tailed paired Student's t test (F) and one-way ANOVA with Dunnett's multiple comparisons test (G, H, K-N). The data are shown as the means ± SDs. * $p < 0.05$.

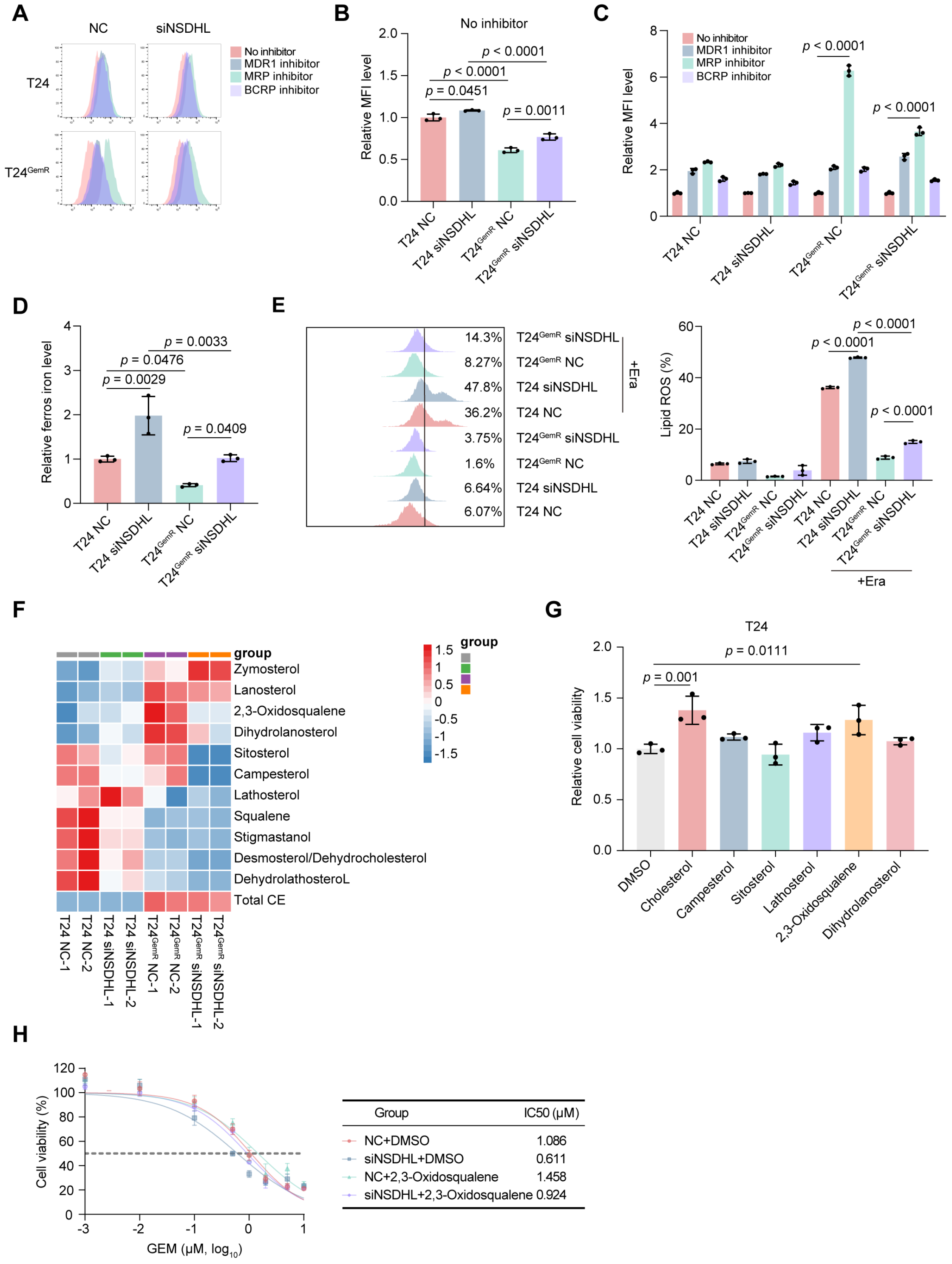

Supplementary Figure S4. Downstream functional consequences of NSDHL-dependent cholesterol accumulation in GEM-resistant bladder cancer cells.

(A) EFLUXX-ID Green assay measuring the contribution of individual ABC transporters to efflux activity in T24 and T24^{GemR} cells with or without NSDHL knockdown. **(B)** Statistical analysis of intracellular mean fluorescence intensity (MFI) in T24 and T24^{GemR} cells without inhibitors. **(C)** Statistical analysis of the contribution

of each individual ABC transporter protein to efflux activity in T24 and T24^{GemR} cells with or without NSDHL knockdown. **(D)** Relative intracellular ferrous iron (Fe^{2+}) levels in T24 and T24^{GemR} cells with or without NSDHL knockdown. **(E)** Representative flow cytometry plots (left) and statistical graph (right) of lipid ROS in T24 cells and T24^{GemR} cells with or without NSDHL knockdown. **(F)** Heatmap of targeted sterol intermediates in T24 and T24^{GemR} cells with or without NSDHL knockdown. **(G)** Relative cell viability of T24 cells in the indicated group. Relative cell viability was calculated as: OD (sterol + GEM) / OD (sterol alone). **(H)** Cell viability and IC50 values of T24 cells in the indicated groups after 48 hrs of treatment with different concentrations of GEM, as determined via the MTT assay (n = 5). Statistical significance was determined by one-way ANOVA with Tukey's multiple comparisons test (B-E) and one-way ANOVA with Dunnett's multiple comparisons test (G). The data are shown as the means ± SDs.

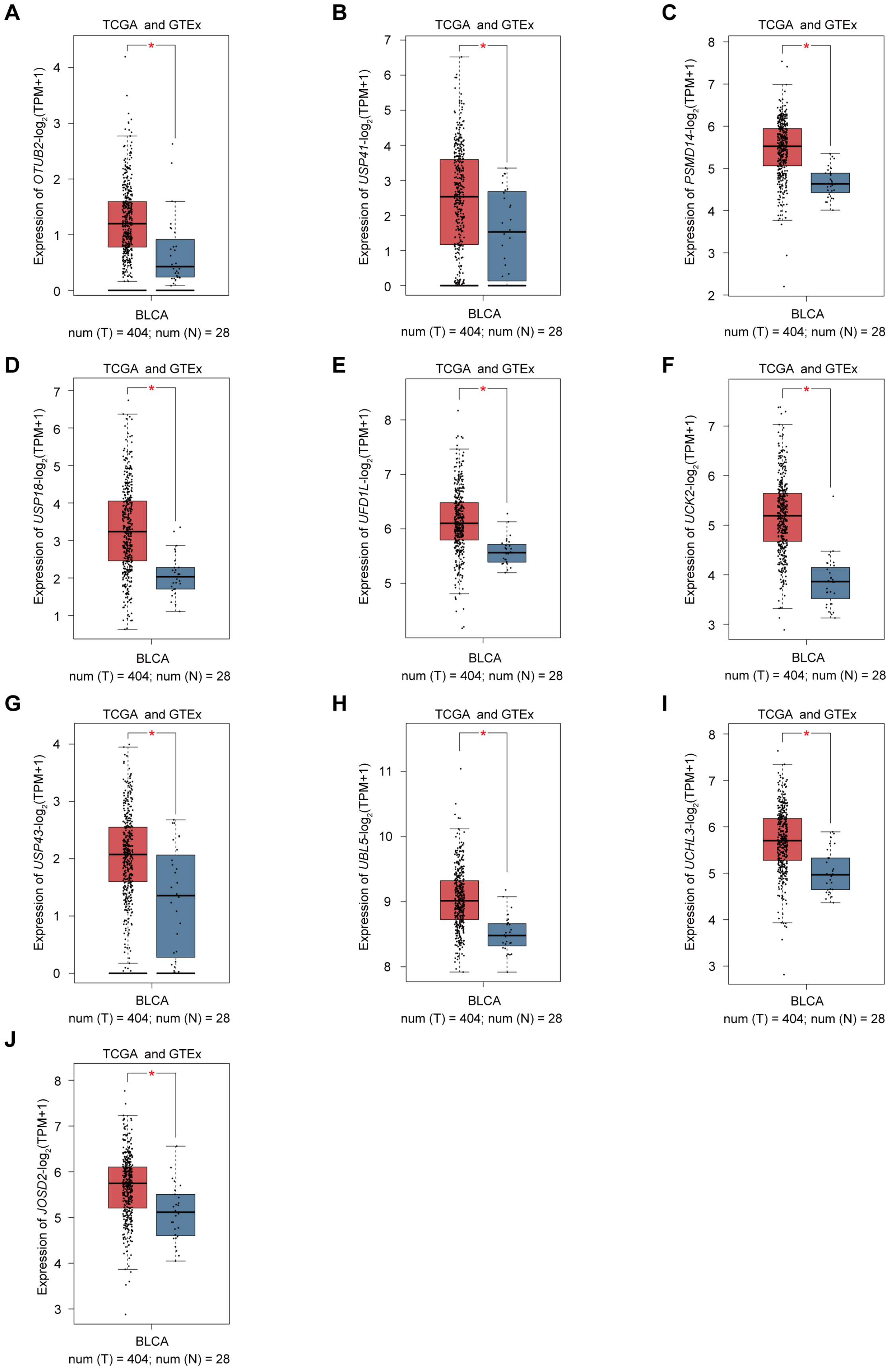

Supplementary Figure S5. Ten deubiquitinases are highly expressed in BLCA.

(A-J) Expression levels of ten DUBs in BLCA and normal tissue at GEPIA. |Log_2FC| Cutoff: 0.5, p-value Cutoff: 0.05. * $p < 0.05$.

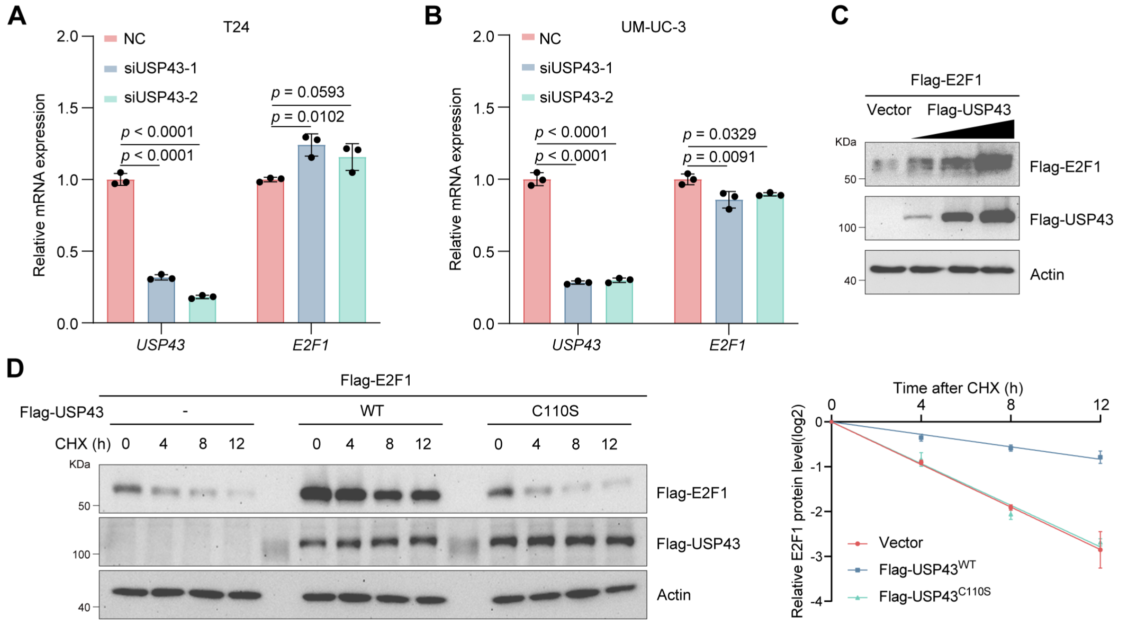

Supplementary Figure S6. USP43 stabilizes E2F1 through deubiquitination.

(A-B) After USP43 was knocked down in T24 (A) and (B) UM-UC-3 cells, the mRNA level was detected via qRT-PCR (n = 3). **(C)** Flag-E2F1 was cotransfected with empty vector or increasing concentrations of Flag-USP43 into 293T cells, and then Flag-E2F1 was detected by subsequent immunoblot analysis. **(D)** Representative Western blot images (left panel) and statistical results (right panel) of E2F1 after Flag-E2F1 was cotransfected with empty vector or with Flag-USP43 (wild-type or C110S) into 293T cells. The cells were treated with 50 μg/mL CHX and subsequently harvested at the indicated time points (n = 3). Statistical significance was determined by one-way ANOVA with Dunnett's multiple comparisons test (A, B). The data are shown as the means ± SDs.

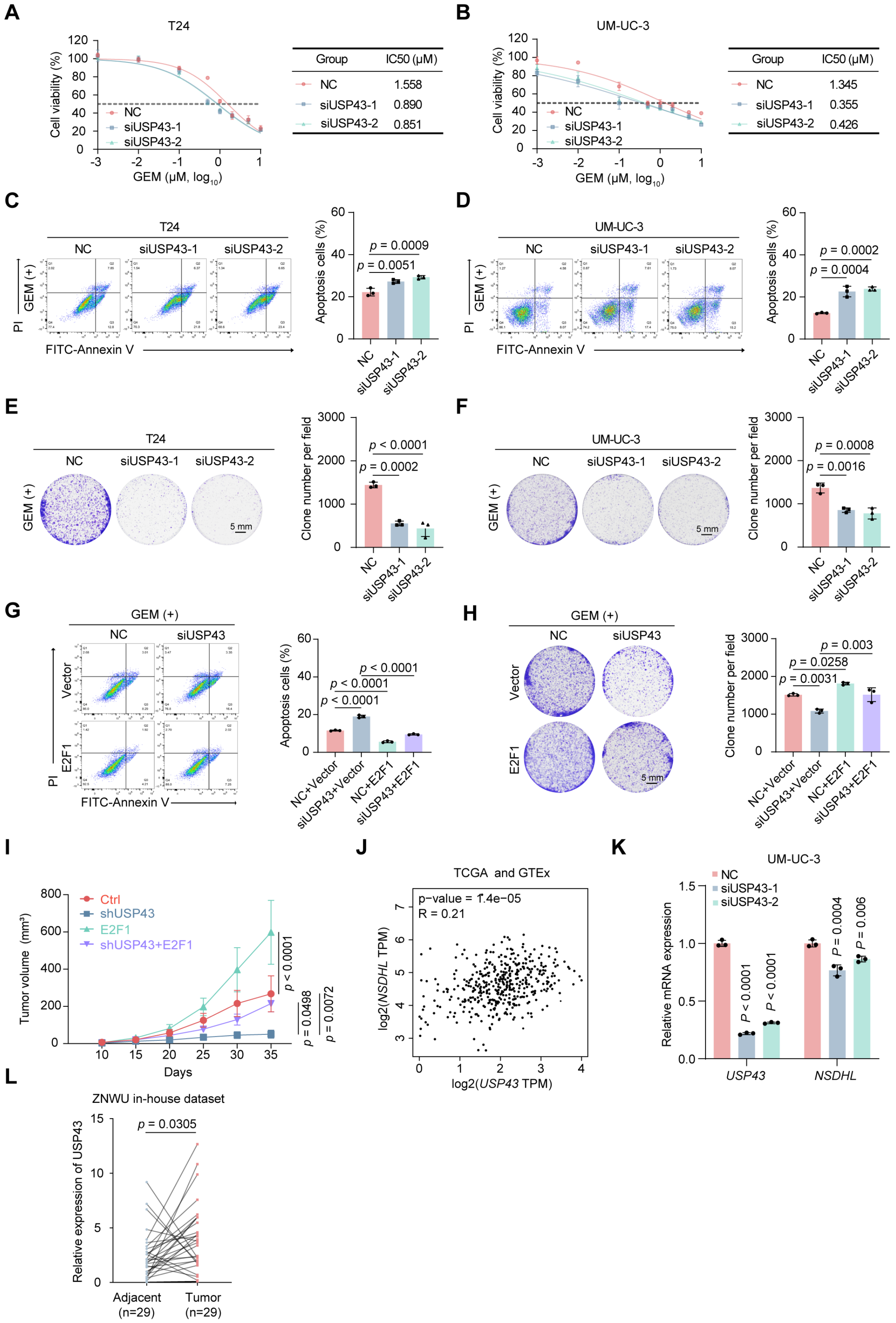

Supplementary Figure S7. USP43 promotes gemcitabine resistance in BLCA *in vitro*.

(A-B) After USP43 was knocked down, the viability and IC50 values of T24 (A) and UM-UC-3 (B) cells after 48 hrs of exposure to various concentrations of GEM were measured via the MTT assay (n = 5). **(C-D)** Representative images (left panel) and statistical analysis (right panel) of apoptosis assays in T24 (C) and UM-UC3 (D) cells with USP43 knockdown, followed by 1 μM GEM treatment for 48 hrs (n = 3). **(E-F)** Representative images (left panel) and statistical analysis (right panel) of colony formation assays in T24 cells (E) or UM-UC3 cells (F) with USP43 knockdown, followed by 1 μM GEM treatment (n = 3). **(G)** Apoptosis analysis of T24 cells in the indicated groups after 48 hrs of treatment with 1 μM GEM (n = 3). **(H)** Colony formation assay of T24 cells in the indicated groups after 48 hrs of treatment with 1 μM GEM (n = 3). **(I)** The tumor volume of the indicated group was measured on different days (n = 6). **(J)** Pearson's correlation test showing a significant positive association between USP43 and E2F1 mRNA expression in BLCA tumor, BLCA normal and bladder tissues in GEPIA. **(K)** The mRNA levels of USP43 and NSDHL in UM-UC-3 cells were detected via qRT-PCR after USP43 knockdown (n = 3). **(L)** mRNA expression levels of USP43 in BLCA tissues (n = 29) and corresponding adjacent normal tissues (n=29) from the ZNWU in-house dataset. Statistical significance was determined by one-way ANOVA with Dunnett's multiple comparisons test (C-F, K), one-way ANOVA with Tukey's multiple comparisons test (G-I), Pearson's correlation (J) and two-tailed paired Student's t test (L). The data are shown as the means ± SDs.

Supplementary Tables S1-S2

Supplementary Table S1. Primer sequences for qRT-PCR and ChIP-qPCR.

**Primer sequences for qRT-PCR**		
**Gene**	**Forward (5' – 3')**	**Reverse (5' – 3')**
Actin	GATCCACATCTGCTGGAAG	CAGCACAATGAAGATCAAGA
E2F1	CATCCCAGGAGGTCACTTCTG	GACAACAGCGGTTCTTGCTC
ACAT2	GCGGACCATCATAGGTTCCTT	ACTGGCTTGTCTAACAGGATTCT
FASN	AAGGACCTGTCTAGGTTTGATGC	TGGCTTCATAGGTGACTTCCA
HMGCS1	GATGTGGGAATTGTTGCCCTT	ATTGTCTCTGTTCCAACTTCCAG
IDI1	AACACTAACCACCTCGACAAGC	AGACACTAAAAGCTCGATGCAA
NSDHL	CAAGTCGCACGGACTCATTTG	ACTGTGCATCTCTTGGCCTG
CCNE1	GCCAGCCTTGGGACAATAATG	CTTGCACGTTGAGTTTGGGT
USP43	CGCCTGGAAGTGTCCTCAC	CCACCTGGCAGAACCTTTTG
**Primer sequences for ChIP-qPCR**		
**Primer**	**Forward (5' – 3')**	**Reverse (5' – 3')**
NSDHL-P1	GGCTCCATACCATGATAATT	GATAAGTGAAATTGATAAGGAAGG
NSDHL-P2	TTTCGCTGAGAACTTGATGC	ATGGGTGTCTGTAACTATGT
NSDHL-P3	CCCCTGAATGCCCCTGTA	CCCCTGACTCTCTCAGAC
NSDHL-P4	CCGAGCTGGGCCAATCCT	AGCAGGCTGCACCCACTG
LC3B	TGGAGGGGAAAGGATGGTCG	GGGGCGGAGCAGGTGTGTG

qRT-PCR: Quantitative reverse transcription PCR;
ChIP: Chromatin immunoprecipitation.

Supplementary Table S2. Information for primary antibodies used in this study.

Assay	Antibody	Company	Catalog	RRID
WB	Actin	Santa Cruz	sc-47778	AB_626632
	E2F1	CST	3742	AB_2096936
	Cyclin E1	Santa Cruz	sc-377100	AB_2923122
	NSDHL	Abcam	ab190353	AB_3697837
	USP43	Abgent	AP14283b	AB_11135143
	HA-tag	Origene	TA180128	AB_2622290
	Flag-tag	Sigma	F1804	AB_262044
	Myc-tag	ABclonal	AE010	AB_2770408
	GFP-tag	Santa Cruz	sc-9996	AB_627695
IP	E2F1	CST	3742	AB_2096936
	HA-tag	Origene	TA180128	AB_2622290
	Flag-tag	Sigma	F1804	AB_262044
	GFP-tag	Santa Cruz	sc-9996	AB_627695
	IgG	Proteintech	B900610	AB_3674206
IF	GFP-tag	Santa Cruz	sc-9996	AB_627695
	Flag-tag	Sigma	F1804	AB_262044
ChIP	E2F1	CST	3742	AB_2096936
IHC	E2F1	ABclonal	A2067	AB_2764090
	Ki67	Abcam	ab16667	AB_302459
	NSDHL	Abcam	ab190353	AB_3697837

WB: Western blot;
IP: Immunoprecipitation;
IF: Immunofluorescence;
ChIP: Chromatin immunoprecipitation;
IHC: Immunohistochemistry.
